# A new copper complex of 1-(1*H*-benzo[*d*]imidazol-2-yl)guanidine on magnetic Fe_3_O_4_ nanoparticles as a green, reusable, robust and homoselective nanocatalyst in the synthesis of tetrahydrobenzo[*b*]pyrans

**DOI:** 10.1039/d6ra02796b

**Published:** 2026-07-21

**Authors:** Bahman Tahmasbi, Abdulkhaleq Ali Hussein, Saeid Taghavi Fardood, Omid Soleimani

**Affiliations:** a Department of Chemistry, Faculty of Science, Ilam University P. O. Box 69315516 Ilam Iran b.tahmasbi@ilam.ac.ir bah.tahmasbi@gmail.com

## Abstract

In this work, magnetic Fe_3_O_4_ nanoparticles were encapsulated using a silica layer *via* a sol–gel method, giving SiO_2_@Fe_3_O_4_. Then, the surface of the SiO_2_@Fe_3_O_4_ was modified using 3-iodopropyltrimethoxysilane (3-IPTMS), giving IPTMS@SiO_2_@Fe_3_O_4_. Additionally, 1-(1*H*-benzo[*d*]imidazol-2-yl)guanidine ligand (BimG) was synthesized from the condensation of cyanoguanidine and benzene-1,2-diamine under acidic conditions. Then, a new copper complex of BimG was anchored on the surface of IPTMS@SiO_2_@Fe_3_O_4_ to produce Cu-BimG@SiO_2_@Fe_3_O_4_. The obtained nanocatalyst was characterized using TGA, BET, WDX, EDS, FTIR, XRD, SEM, AAS, and VSM. Cu-BimG@SiO_2_@Fe_3_O_4_ was investigated as an effective, homoselective and reusable nanocatalyst for synthesizing tetrahydrobenzo[*b*]pyrans through a multicomponent reaction. The homoselectivity of this catalyst was confirmed by NMR spectroscopy. This Cu-BimG@SiO_2_@Fe_3_O_4_ catalyst showed good reusability over several runs without a meaningful loss of catalytic performance. The recovered Cu-BimG@SiO_2_@Fe_3_O_4_ nanocatalyst was characterized by SEM, EDS, WDX, and FTIR techniques, which matched with the fresh catalyst.

## Abstract 2

In this work, a copper complex of 1-(1*H*-benzo[*d*]imidazol-2-yl)guanidine was immobilized on the surface of Fe_3_O_4_ MNPs as an effective, homoselective and reusable nanocatalyst for the synthesizing of tetrahydrobenzo[*b*]pyrans.

## Graphical abstract

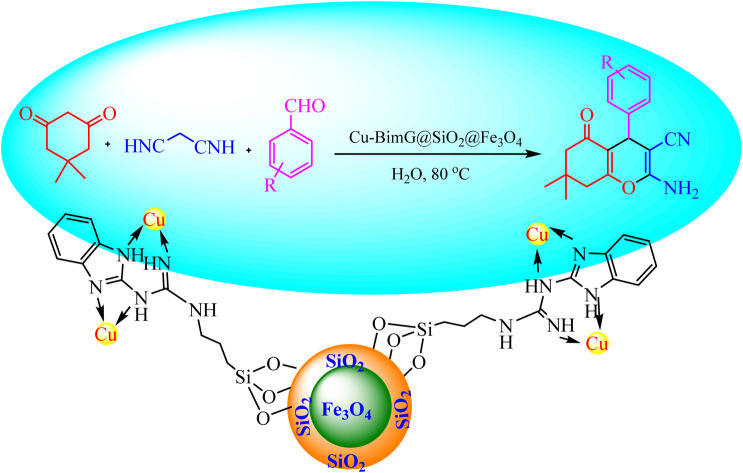

## Introduction

Nanotechnology has grown dramatically since 1959.^1^ Materials with sizes between 1 and 100 nanometers (nm) are classified as nanoparticles.^2^ Nanoparticles (NPs) have different chemical, catalytic, physical, and electrical properties depending on their morphology, size, and phases, and for this reason they are of interest to scientists in various fields.^3–5^ Fe_3_O_4_ (or magnetite) NPs have found many applications due to their unique properties, *e.g.*, biosensing,^6^ energy storage,^7^ magnetic fluids,^8^ biotechnology^9,10^ microwave absorption,^11,12^ catalysis,^4,13–15^ and lithium-ion batteries.^16,17^ Nanomagnetite can also be used to absorb metal ions from the environment.^18,19^ Nanomagnetite is obtained in various shapes: cubic, spherical, planar, concave, octahedral, rod, and branch.^3^ Also, Fe_3_O_4_ NPs can be prepared by various methods including microwave, co-precipitation, non-hydrolytic, sonochemical, and thermal solvent.^20^ So far, magnetic Fe_3_O_4_ nanoparticles have been widely used for the preparation of nanocatalysts.^21–25^ Fe_3_O_4_-based nanocatalysts have excellent catalytic activity and are easily separated due to their magnetic properties.^26,27^ Fe_3_O_4_-based nanocatalysts have been used in various organic processes, *e.g.*, oxidation reactions, reduction reactions, condensation reactions, coupling reactions, multicomponent reactions (MCRs), cycloaddition, and protection/deprotection reactions.^14,28–33^ Among the organic reactions, MCRs are a class of reactions in which two or more starting materials react with each other to produce a single product.^29,34,35^ Since Strecker first introduced multicomponent reactions for the synthesis of amino acids 175 years ago, this has been the basis for the synthesis of a wide range of different compounds.^36,37^ A wide range of reactions have been built on this basis, such as: Biginelli reaction,^38^ Mannich reaction,^39^ Hantzsch reaction,^40,41^ Bucherer–Bergs reaction,^42^ Asinger reaction,^43^ Passerini reaction,^44^ Gewald reaction,^45^ Grieco three-component coupling,^46^ Kabachnik–Fields reaction,^47^ and Ugi reaction.^48^ MCRs are of great interest due to their high efficiency and facile production of various products. In multicomponent reactions, the first two compounds react together, and then the next component reacts with the product of the first stage.^49^ The mechanism of MCRs is usually reversible in the early stages, but irreversible in the final stage, where the product is formed.^49^ The type of catalyst, solvent, temperature, and optimization of reaction conditions have a direct impact on the performance of multicomponent reactions. However, these types of reactions are of great importance due to their shorter reaction time, high efficiency, easy separation of products, and milder reaction environment.^50^ MCRs are very efficient for the synthesis and further development of a wide range of biological and pharmaceutical compounds.^50^ One of the most important pharmaceutical species prepared through multicomponent reactions is tetrahydrobenzo[*b*]pyrans derivatives.^51^ Tetrahydrobenzo[*b*]pyrans are compounds from the pyran family,^52^ which are synthesized from a three-component reaction of aldehyde, malononitrile, and dimedone.^53–55^ Pyran derivatives have many pharmacological activities, including anti-Parkinson's,^56^ anticancer,^57^ diuretic,^58^ spasmolytic,^59^ antioxidant,^60^ and anti-HIV effects .^61^ Other applications of tetrahydrobenzo[*b*]pyrans include their use as intermediates for the preparation of other biologically active compounds, such as pyrano[2,3-*c*]pyrazoles,^62^ polyazanaphthalenes,^62^ pyrano[2,3-*b*]pyridine,^62^ and pyridin-2-ones.^63^ Because of these important applications, researchers have proposed various methods for the preparation of tetrahydrobenzo[*b*]pyrans using various catalysts, *e.g.* potassium phosphate,^64^ Fe_3_O_4_@hydrol-PMMAn,^65^ LDH@PTRMS@NDBD@CuI,^66^ sodium stearate,^67^ g-C_3_N_4_/Fe_3_O_4_@P_2_W_15_V_3_,^68^ Fe_3_O_4_@SiO_2_@SO_3_H,^69^ LaCoO_3_/Co_3_O_4_,^70^ Fe_3_O_4_@RHA@TiO_2_,^71^ UiO-66-NH_2_-BBG@Cu(ii),^72^ and CoFe_2_O_4_@SiO_2_-CPTES-melamine-Cu.^73^ In this study, we present a reliable and efficient method for the preparation of tetrahydrobenzo[*b*]pyrans using Cu-BimG@SiO_2_@Fe_3_O_4_ nanocatalyst. Due to its magnetic properties, the Cu-BimG@SiO_2_@Fe_3_O_4_ nanocatalyst is easily separated from the product and can be recycled and reused several times. This is the first report of the synthesis and characterization of Cu-BimG@SiO_2_@Fe_3_O_4_ nanocatalyst and its application in organic reactions. More importantly, this catalyst shows good homoselectivity in the synthesis of tetrahydrobenzo[*b*]pyran derivatives in an aqueous environment, which increases the novelty of the work.

## Experimental

### Preparation of magnetic Fe_3_O_4_ nanoparticles

FeCl_3_·6H_2_O (17.514 g) and FeCl_2_·4H_2_O (6.41 g) were placed in a 500 mL beaker at 80 °C and stirred under a nitrogen atmosphere. Then, under the same conditions, 30 mL of aqueous ammonia solution 25% was added to obtain a black suspension. The reaction vessel was then stirred under the same conditions for 30 minutes. Finally, the contents of the reaction vessel were cooled to room temperature, and the black precipitate was separated using an external magnet, washed several times with distilled water, and dried at room temperature.

### Encapsulation of Fe_3_O_4_ with SiO_2_

First, 2 g of magnetic Fe_3_O_4_ nanoparticles was dispersed in 40 mL of water for 30 minutes under ultrasonic conditions. Next, 100 mL of ethanol was added to the reaction medium. Then, while stirring at room temperature, 5.36 g of polyethylene glycol 400 (PEG-400), 10 mL of ammonia solution, and 2 mL of tetraethyl orthosilicate (TEOS) were added. The resulting mixture was stirred at room temperature for 30 hours. Finally, the obtained SiO_2_@Fe_3_O_4_ was washed with ethanol and distilled water, then dried at room temperature.

### Preparation of 3-iodopropyltrimethoxysilan (3-IPTMS)

**Scheme 1:**
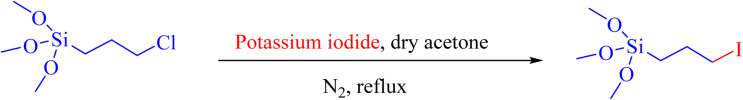
Preparation of 3-iodopropyltrimethoxysilane (3-IPTMS).

In a 50 mL flask, a mixture of potassium iodide (20 mmol) was dissolved in 50 mL of dry acetone and 3-chloropropyltrimethoxysilane (20 mmol) under stirring. The mixture was refluxed under a nitrogen atmosphere for 30 h (Scheme 1). 3-IPTMS was isolated by simple filtration as a yellow liquid after evaporation of acetone.

### Preparation of the 1-(1*H*-benzo[*d*]imidazol-2-yl)guanidine ligand (BimG)

**Scheme 2:**
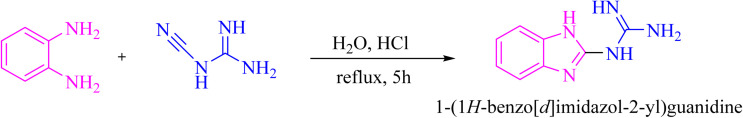
Preparation of 1-(1*H*-benzo[*d*]imidazol-2-yl)guanidine.

A mixture of cyanoguanidine (4 mmol), benzene-1,2-diamine (4 mmol), and 4 mL of hydrochloric acid (HCl) in 20 mL of distilled water was stirred and refluxed for 5 hours (Scheme 2). After the reaction was completed, the resulting mixture was cooled to room temperature, and sodium hydroxide (NaOH) was slowly added to the reaction mixture until the product formed as a precipitate. Finally, the 1-(1*H*-benzo[*d*]imidazol-2-yl)guanidine precipitate was isolated by filtration.

### Preparation of Cu-BimG@SiO_2_@Fe_3_O_4_

**Scheme 3:**
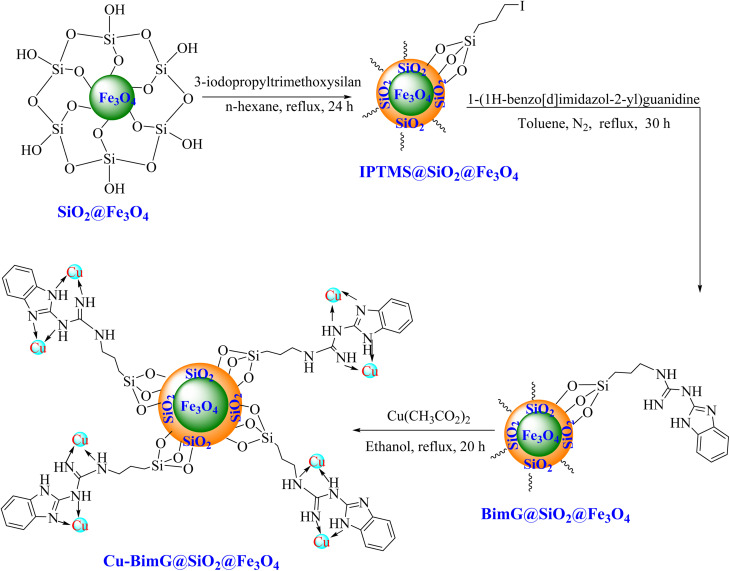
Preparation of Cu-BimG@SiO_2_@Fe_3_O_4_.

In a 100 mL flask, 1 g of SiO_2_@Fe_3_O_4_ nanoparticles was dispersed in 30 mL of *n*-hexane using an ultrasonic bath. Next, 3-IPTMS (1.5 mL) was added, and the resulting mixture was refluxed for 24 h. When the reaction was completed, the magnetic IPTMS@SiO_2_@Fe_3_O_4_ nanoparticles were separated using an external magnet and washed with ethanol several times. Then, 1 g of IPTMS@SiO_2_@Fe_3_O_4_ was dispersed in toluene solvent and 1 mmol of the 1-(1*H*-benzo[*d*]imidazol-2-yl)guanidine ligand was added. The resulting mixture was stirred under a nitrogen atmosphere and refluxed for 30 hours. Then, the reaction mixture was cooled to room temperature and the product BimG@SiO_2_@Fe_3_O_4_ was separated by an external magnet and washed several times with ethanol. Finally, 1 g of magnetic BimG@SiO_2_@Fe_3_O_4_ nanoparticles was dispersed in ethanol solvent for 30 minutes, then 2 mmol of Cu(CH_3_CO_2_)_2_ was added and the resulting mixture was refluxed and stirred for 20 hours (Scheme 3). The resulting mixture was cooled to room temperature, then the magnetic Cu-BimG@SiO_2_@Fe_3_O_4_ nanocatalyst was separated by an external magnet and washed several times with ethanol and distilled water.

### General method for the preparation of tetrahydrobenzo[*b*]pyrans catalyzed by Cu-BimG@SiO_2_@Fe_3_O_4_

**Scheme 4:**
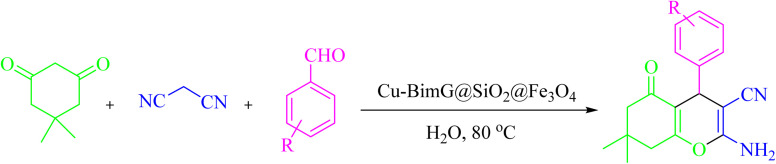
Preparation of tetrahydrobenzo[*b*]pyran catalyzed by Cu-BimG@SiO_2_@Fe_3_O_4_.

A mixture of aldehyde (0.5 mmol), malononitrile (0.5 mmol), dimedone (0.5 mmol) and Cu-BimG@SiO_2_@Fe_3_O_4_ (15 mg) as a catalyst was stirred at 80 °C in water (Scheme 4). The progress of the reaction was monitored using thin-layer chromatography (TLC) (in *n*-hexane and ethyl acetate). After the reaction was completed, the resulting mixture was cooled to room temperature, then the aqueous solvent was decanted, next the catalyst was separated using an external magnet, and the product was filtered with 2 mL of dimethyl sulfoxide (DMSO) solvent. A few drops of distilled water were added to the filtered solution to form a precipitate. The resulting precipitate was washed with distilled water and then purified by recrystallization.

### Spectral data

#### 2-Amino-4-(4-chlorophenyl)-7,7-dimethyl-5-oxo-5,6,7,8-tetrahydro-4*H*-chromene-3-carbonitrile

^1^H NMR (250 MHz, DMSO_d6_): *δ*_H_ = 7.35–7.32 (d, *J* = 7.5 Hz, 2H), 7.13–7.16 (d, *J* = 7.5 Hz, 2H), 7.06 (br, 2H), 4.17 (s, 1H), 2.49 (s, 2H), 2.27–2.20 (d, *J* = 17.5 Hz, 1H), 2.11–2.05 (d, *J* = 15 Hz, 1H), 1.01 (s, 3H), 0.93 (s, 3H) ppm.

IR (KBr) cm^−1^: 3380, 3182, 2958, 2889, 2188, 1677, 1634, 1604, 1490, 1412, 1365, 1309, 1286, 1247, 1216, 1162, 1139, 1093, 1032, 1014, 973, 939, 917, 853, 828, 769, 682, 619, 562, 520, 472.

#### 2-Amino-7,7-dimethyl-4-(3-nitrophenyl)-5-oxo-5,6,7,8-tetrahydro-4*H*-chromene-3-carbonitrile

^1^H NMR (250 MHz, DMSO_d6_): *δ*_H_ = 7.80–7.78 (d, *J* = 5 Hz, 1H), 7.64 (s, 1H), 7.43–7.31 (m, 2H), 7.19 (br, 2H), 4.90 (s, 1H), 2.48 (s, 2H), 2.21–2.15 (d, *J* = 15 Hz, 1H), 2.02–1.95 (d, *J* = 17.5 Hz, 1H), 0.99 (s, 3H), 0.85 (s, 3H) ppm.

IR (KBr) cm^−1^: 3476, 3332, 3253, 3206, 2960, 2870, 2836, 2198, 1686, 1662, 1597, 1526, 1468, 1414, 1362, 1254, 1214, 1143, 1041, 976, 917, 861, 826, 785, 737, 703, 677, 644, 562, 518, 434.

#### 2-Amino-4-(4-methoxyphenyl)-7,7-dimethyl-5-oxo-5,6,7,8-tetrahydro-4*H*-chromene-3-carbonitrile

^1^H NMR (250 MHz, DMSO_d6_): *δ*_H_ = 7.03–6.94 (m, 4H), 6.83–6.79 (m, 2H), 4.09 (s, 1H), 3.68 (s, 3H), 2.48 (s, 2H), 2.26–2.19 (d, *J* = 17.5 Hz, 1H), 2.09–2.03 (d, *J* = 15 Hz, 1H), 1.00 (s, 3H), 0.92 (s, 3H) ppm.

IR (KBr) cm^−1^: 3374, 3324, 3257, 3186, 3011, 2964, 2895, 2833, 2193, 1685, 1656, 1605, 1509, 1463, 1414, 1369, 1324, 1300, 1252, 1213, 1164, 1139, 1033, 973, 914, 843, 804, 774, 721, 696, 648, 627, 568, 525.

#### 2-Amino-4-(3-hydroxyphenyl)-7,7-dimethyl-5-oxo-5,6,7,8-tetrahydro-4*H*-chromene-3-carbonitrile

^1^H NMR (250 MHz, DMSO_d6_): *δ*_H_ = 9.30 (br, 1H), 7.06–6.96 (m, 3H), 6.54–6.52 (m, 3H), 4.03 (s, 1H), 2.48 (s, 2H), 2.27–2.20 (d, *J* = 17.5 Hz, 1H), 2.11–2.05 (d, *J* = 15 Hz, 1H), 1.01 (s, 3H), 0.94 (s, 3H) ppm.

^13^C NMR (100 MHz, DMSO_d6_): *δ*_C_ = 195.5, 162.3, 158.5, 157.3, 146.1, 129.1, 119.6, 117.8, 114.1, 113.6, 112.9, 58.6, 50.1, 35.5, 31.7, 28.4, 26.8 ppm.

IR (KBr) cm^−1^: 3457, 3312, 3207, 2966, 2879, 2199, 1681, 1643, 1595, 1481, 1375, 1338, 1287, 1257, 1215, 1146, 1079, 1040, 975, 953, 915, 871, 817, 798, 767, 705, 657, 616, 562, 516, 469.

#### 2-Amino-4-(4-(dimethylamino)phenyl)-7,7-dimethyl-5-oxo-5,6,7,8-tetrahydro-4*H*-chromene-3-carbonitrile

^1^H NMR (250 MHz, DMSO_d6_): *δ*_H_ = 6.90–6.89 (m, 4H), 6.61 (br, 2H), 4.01 (s, 1H), 2.47 (s, 2H), 2.25–2.18 (d, *J* = 17.5 Hz, 1H), 2.08–2.01 (d, *J* = 17.5 Hz, 1H), 1.00 (s, 3H), 0.92 (s, 3H) ppm.

#### 2-Amino-4-(3-bromophenyl)-7,7-dimethyl-5-oxo-5,6,7,8-tetrahydro-4*H*-chromene-3-carbonitrile

^1^H NMR (250 MHz, DMSO_d6_): *δ*_H_ = 7.39–7.22 (m, 3H), 7.15–7.09 (m, 3H), 4.18 (s, 1H), 2.49 (s, 2H), 2.28–2.21 (d, *J* = 17.5 Hz, 1H), 2.13–2.06 (d, *J* = 17.5 Hz, 1H), 1.01 (s, 3H), 0.94 (s, 3H) ppm.

IR (KBr) cm^−1^: 3345, 3259, 3165, 2964, 2192, 1685, 1656, 1604, 1467, 1415, 1371, 1302, 1251, 1215, 1160, 1139, 1068, 1036, 974, 881, 814, 791, 767, 713, 694, 649, 611, 567, 464, 435.

#### 2-Amino-7,7-dimethyl-5-oxo-4-phenyl-5,6,7,8-tetrahydro-4*H*-chromene-3-carbonitrile

^1^H NMR (250 MHz, DMSO_d6_): *δ*_H_ = 7.27–7.24 (d, *J* = 7.5 Hz, 2H), 7.19–7.10 (m, 3H), 7.00 (br, 2H), 4.15 (s, 1H), 2.50 (s, 2H), 2.27–2.21 (d, *J* = 15 Hz, 1H), 2.11–2.05 (d, *J* = 15 Hz, 1H), 1.02 (s, 3H), 0.94 (s, 3H) ppm.

IR (KBr) cm^−1^: 3396, 3325, 3252, 3212, 3028, 2964, 2883, 2825, 2199, 1682, 1660, 1603, 1492, 1452, 1413, 1370, 1249, 1214, 1159, 1138, 1035, 972, 887, 838, 815, 786, 737, 696, 652, 580, 560, 530, 495, 422.

#### 2-Amino-4-(4-formylphenyl)-7,7-dimethyl-5-oxo-5,6,7,8-tetrahydro-4*H*-chromene-3-carbonitrile

^1^H NMR (300 MHz, DMSO_d6_): *δ*_H_ = 9.93 (s, 1H), 7.84–7.81 (d, *J* = 9 Hz, 2H), 7.37–7.35 (d, *J* = 6 Hz, 2H), 7.14 (br, 2H), 4.27 (s, 1H), 2.52 (s, 2H), 2.27–2.22 (d, *J* = 15 Hz, 1H), 2.11–2.06 (d, *J* = 15 Hz, 1H), 1.02 (s, 3H), 0.94 (s, 3H) ppm.

IR (KBr) cm^−1^: 3408, 3332, 3257, 3212, 2962, 2934, 2872, 2193, 1686, 1602, 1575, 1465, 1414, 1396, 1367, 1321, 1250, 1213, 1144, 1040, 973, 916, 851, 819, 796, 773, 694, 623, 562, 507.

#### 2-Amino-7,7-dimethyl-4-(2-nitrophenyl)-5-oxo-5,6,7,8-tetrahydro-4*H*-chromene-3-carbonitrile

^1^H NMR (300 MHz, DMSO_d6_): *δ*_H_ = 8.08–8.05 (d, *J* = 9 Hz, 1H), 7.95 (s, 1H), 7.66–7.57 (m, 2H), 7.20 (br, 2H), 4.40 (s, 1H), 2.53 (s, 2H), 2.28–2.23 (d, *J* = 15 Hz, 1H), 2.12–2.07 (d, *J* = 15 Hz, 1H), 1.03 (s, 3H), 0.94 (s, 3H) ppm.

^13^C NMR (100 MHz, DMSO_d6_): *δ*_C_ = 195.6, 163.1, 158.7, 147.8, 146.9, 134.1, 129.9, 121.7, 121.6, 119.2, 111.9, 57.4, 49.9, 35.5, 31.8, 28.3, 26.8 ppm.

IR (KBr) cm^−1^: 3471, 3334, 3256, 3211, 3077, 2961, 2870, 2194, 1689, 1663, 1598, 1525, 1468, 1447, 1412, 1360, 1254, 1212, 1162, 1143, 1042, 977, 946, 917, 861, 827, 784, 735, 699, 675, 644, 610, 561, 514, 455.

## Results and discussion

### Characterization of Cu-BimG@SiO_2_@Fe_3_O_4_

**Fig. 1:**
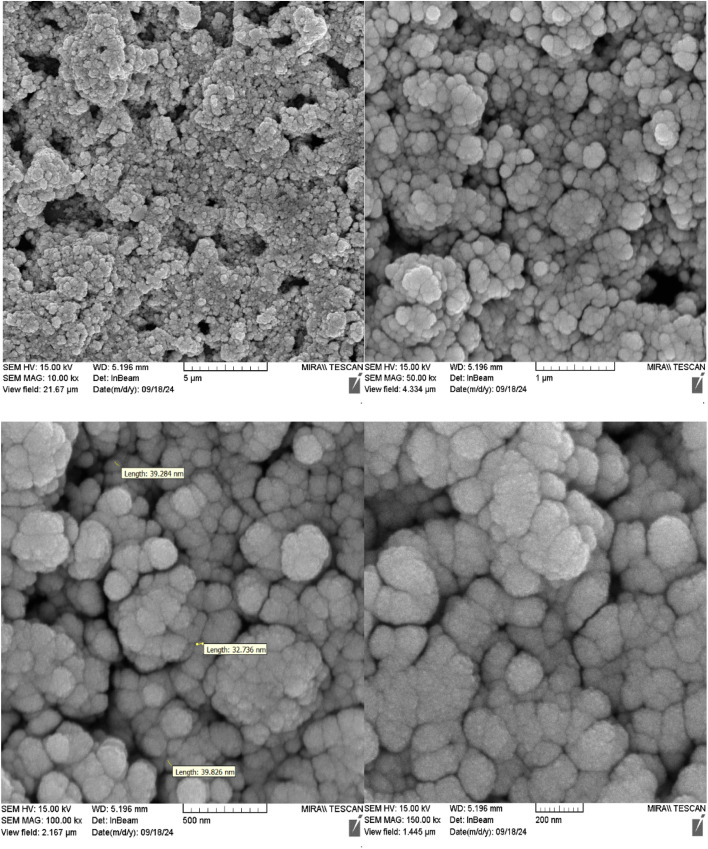
SEM images of the Cu-BimG@SiO_2_@Fe_3_O_4_ nanocatalyst.

**Fig. 2:**
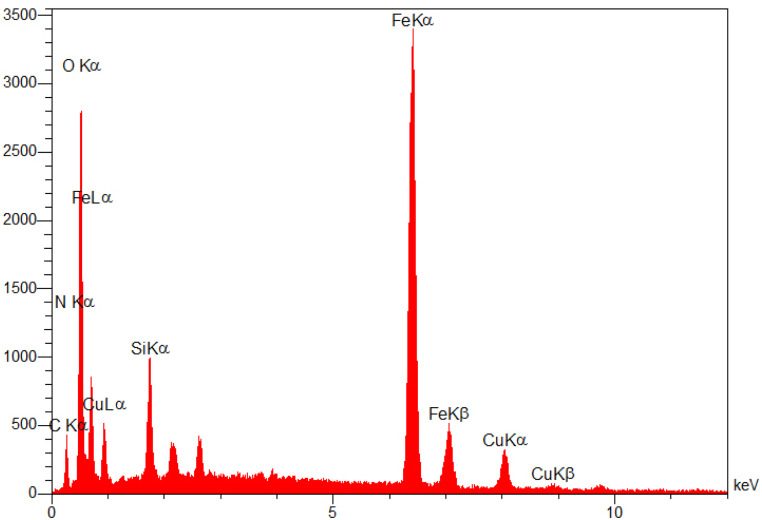
EDS spectrum of the Cu-BimG@SiO_2_@Fe_3_O_4_ nanocatalyst.

Scanning electron microscopy (SEM) images of the Cu-BimG@SiO_2_@Fe_3_O_4_ nanocatalyst (Fig. 1) show spherical and uniform particle morphology with a size of less than 100 nm. Furthermore, the presence of Cu, Fe, N, C, O, and Si elements was confirmed using energy-dispersive X-ray spectroscopy (EDS) analysis, indicating the successful synthesis of the Cu-BimG@SiO_2_@Fe_3_O_4_ nanocatalyst (Fig. 2).

**Fig. 3:**
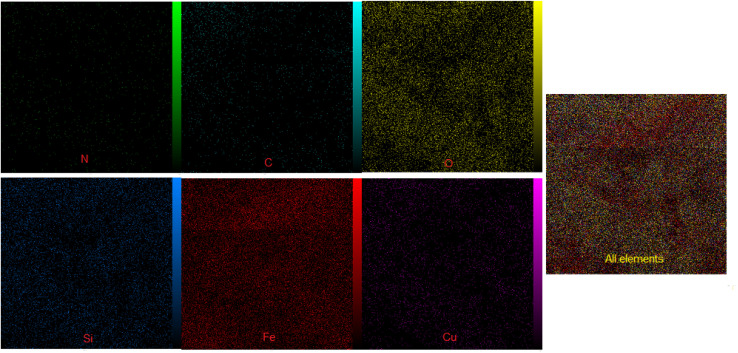
Elemental mapping of the Cu-BimG@SiO_2_@Fe_3_O_4_ nanocatalyst.

The uniform dispersion and distribution of Cu, Fe, N, C, O, and Si is clearly visible in the wavelength-dispersive X-ray spectroscopy (WDX) images (Fig. 3). This uniform distribution indicates that the surface of the Fe_3_O_4_ nanoparticles is well covered by the elements of interest.

Also, the amount of Cu loaded in the Cu-BimG@SiO_2_@Fe_3_O_4_ nanocatalyst was determined to be 0.1 mol g^−1^ using atomic absorption spectroscopy (AAS) analysis.

**Fig. 4:**
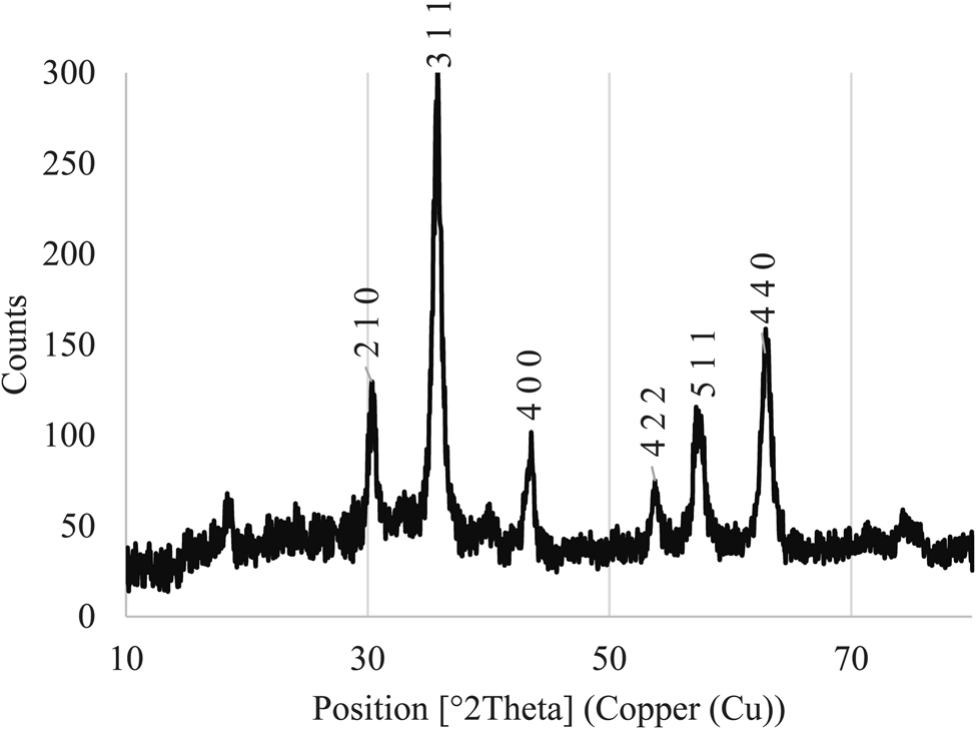
XRD pattern of the Cu-BimG@SiO_2_@Fe_3_O_4_ nanocatalyst.

The crystal structure of the Cu-BimG@SiO_2_@Fe_3_O_4_ catalyst was investigated using X-ray diffraction (XRD). The XRD pattern shows several peaks at 2*θ* = 30.5° (210), 35.7° (311), 43.5° (400), 53.7° (422), 57.2° (511), and 63.2° (440). This pattern corresponds to the crystal structure of Fe_3_O_4_ nanoparticles (Fig. 4). These results show that the crystal structure of the Fe_3_O_4_ nanoparticles (JCPDS 88-0866) was not damaged during the synthesis of the Cu-BimG@SiO_2_@Fe_3_O_4_ catalyst.^74^

**Fig. 5:**
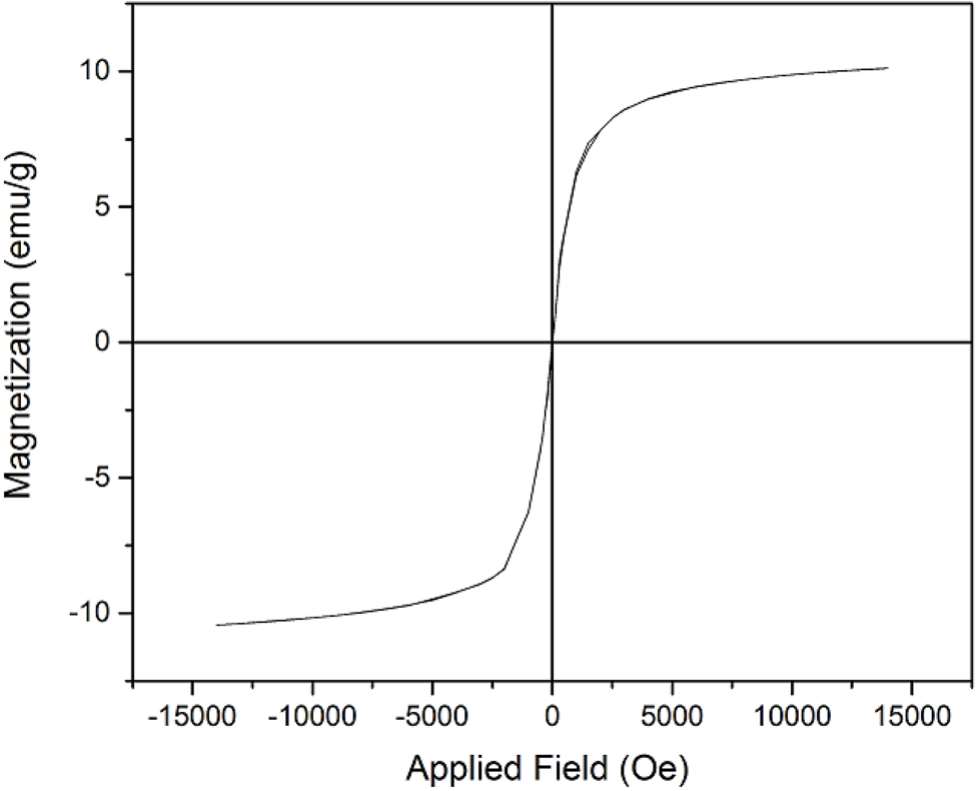
VSM analysis of the Cu-BimG@SiO_2_@Fe_3_O_4_ nanocatalyst.

The magnetic properties of the Cu-BimG@SiO_2_@Fe_3_O_4_ nanocatalyst were investigated at room temperature by vibrating sample magnetometry (VSM; Fig. 5). For the Cu-BimG@SiO_2_@Fe_3_O_4_ nanocatalyst, the magnetic saturation value was 11 emu/g. This low value can be attributed to the overlapping effects of organic groups in the catalyst structure.^75^

**Fig. 6:**
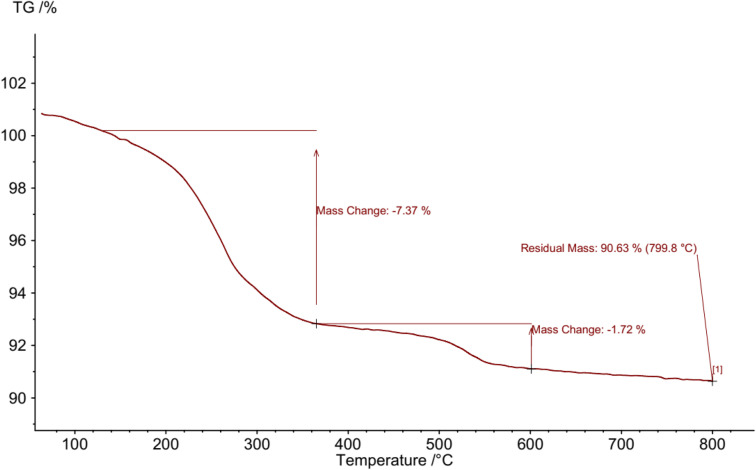
TGA analysis of the Cu-BimG@SiO_2_@Fe_3_O_4_ nanocatalyst.

The thermogravimetric analysis (TGA) of the Cu-BimG@SiO_2_@Fe_3_O_4_ catalyst is shown in Fig. 6. According to this graph, the catalyst experiences a first weight loss of about 7% at 120–360 °C, which is attributed to the removal of organic functional groups. The subsequent weight loss at 500–600 °C of about 1% is related to the decomposition of the Fe_3_O_4_ structure.

**Fig. 7:**
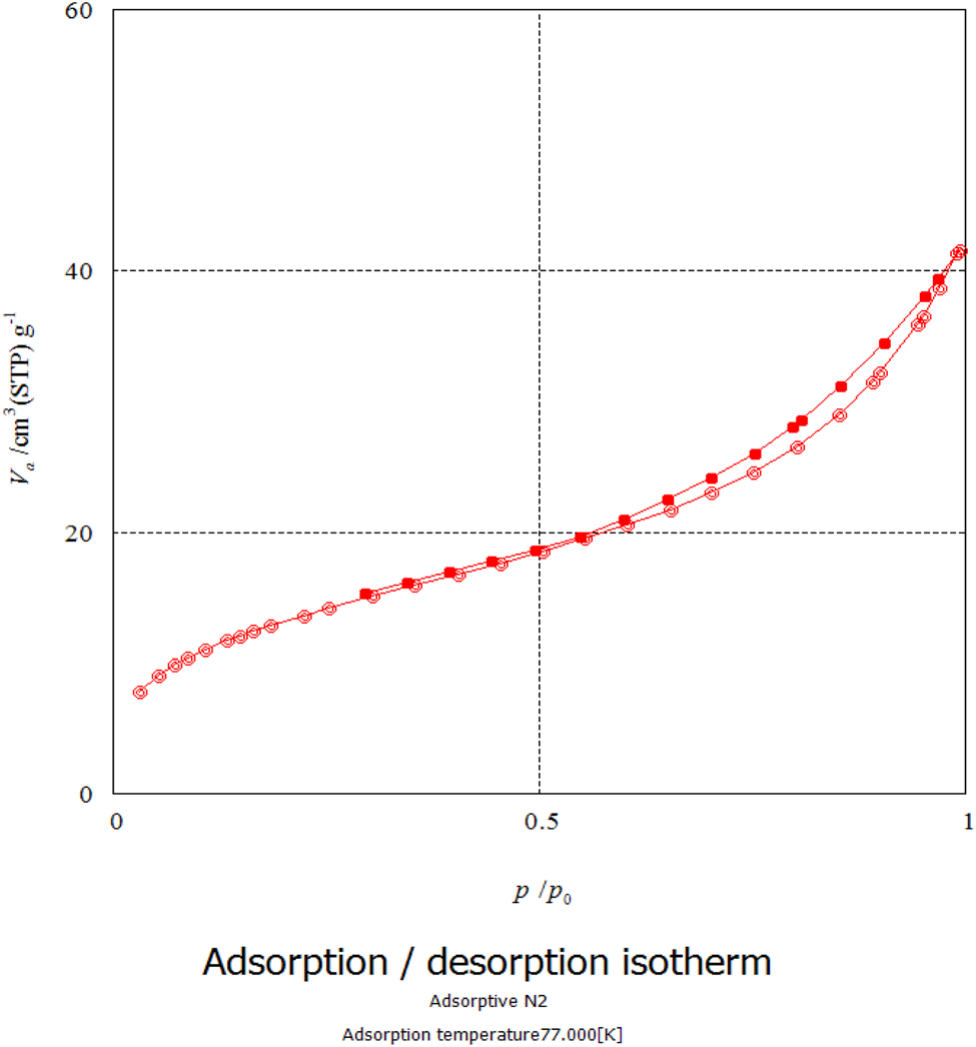
N_2_ adsorption–desorption isotherms for the Cu-BimG@SiO_2_@Fe_3_O_4_ nanocatalyst.

Fig. 7 presents the N_2_ adsorption–desorption isotherms for the Cu-BimG@SiO_2_@Fe_3_O_4_ nanocatalyst can be seen. The average pore diameter, surface area, and total pore volume for the Cu-BimG@SiO_2_@Fe_3_O_4_ nanocatalyst are 5.5704 nm, 45.924 m^2^ g^−1^, and 0.063953 cm^3^ g^−1^, respectively, as shown in Table 1.

**Table 1:** The textural properties of the Cu-BimG@SiO_2_@Fe_3_O_4_ catalyst

Entry	Sample	Pore diameter (nm)	*S*_BET_ (m^2^ g^−1^)	Pore volume (cm^3^ g^−1^)
1	Cu-BimG@SiO_2_@Fe_3_O_4_	5.5704	45.924	0.063953

**Fig. 8:**
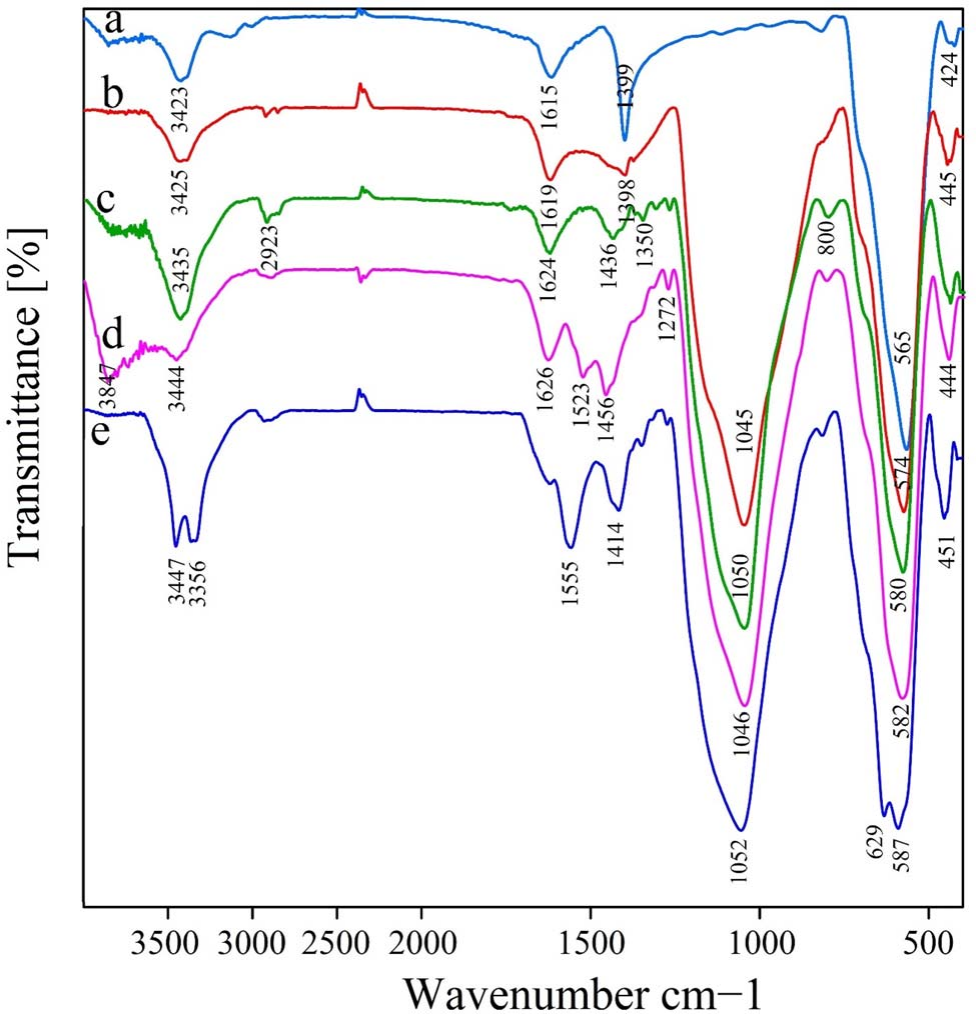
FTIR spectra of (a) Fe_3_O_4_, (b) SiO_2_@Fe_3_O_4_, (c) IPTMS@SiO_2_@Fe_3_O_4_, (d) BimG@SiO_2_@Fe_3_O_4_ and (e) Cu-BimG@SiO_2_@Fe_3_O_4_ nanocatalyst.

Fourier-transform infrared spectroscopy (FTIR) spectra of Fe_3_O_4_, SiO_2_@Fe_3_O_4_, IPTMS@SiO_2_@Fe_3_O_4_, BimG@SiO_2_@Fe_3_O_4_, and Cu-BimG@SiO_2_@Fe_3_O_4_ nanocatalyst are shown in Fig. 8. The peaks in the 424 and 565 cm^−1^ region are attributed to the vibrational modes of the Fe–O bond in the structure of the Fe_3_O_4_ NPs.^28^ In addition, the stretching vibration of the surface OH bond appeared in the 3423–3447 cm^−1^ region and the stretching vibration of the Si–O–Si appeared in the 1045–1052 cm^−1^ region.^76^ The stretching vibration of the Si–O–Si bond was not observed in the FTIR spectrum of Fe_3_O_4_, but it was observed in the FTIR spectrum of SiO_2_@Fe_3_O_4_, indicating the encapsulation of the Fe_3_O_4_ surface by SiO_2_. In the FTIR spectrum of IPTMS@SiO_2_@Fe_3_O_4_, the C–H stretching vibration appeared in the 2923 cm^−1^ region,^77^ indicating the modification of the SiO_2_@Fe_3_O_4_ surface with IPTMS. In the FTIR spectrum of BimG@SiO_2_@Fe_3_O_4_, several peaks appeared in the 1400–1650 cm^−1^ region, which are related to C=C and C=N bonds.^76–78^ Moreover, the stretching vibration of all N–H bonds is seen in the 3440–3850 cm^−1^ region. This evidence indicates that the surface of IPTMS@SiO_2_@Fe_3_O_4_ is successfully functionalized with the BimG ligand. In the FTIR spectrum of Cu-BimG@SiO_2_@Fe_3_O_4_ nanocatalyst, the peaks related to C=N in the 1550–1650 cm^−1^ region are broadened, and the peaks related to N–H in 3440 cm^−1^ appear as bifurcations, indicating the formation of copper complexes with nitrogen atoms that are immobilized on the surface of the Fe_3_O_4_ NPs. The bifurcation of the peak in the 3440 cm^−1^ region indicates that some of the N-groups have formed complexes with copper, and some of the N-groups have remained free due to steric hindrance and have not formed complexes with copper metal. Also, a new peak in the FTIR spectrum of the Cu-BimG@SiO_2_@Fe_3_O_4_ nanocatalyst appears in the 629 cm^−1^ region, which was not seen in the spectra of the previous steps. This peak is probably related to the vibration of the Cu–N bond,^79^ indicating the formation of a copper complex with nitrogen atoms immobilized on the Fe_3_O_4_ nanoparticles.

### Catalytic studies

#### The catalytic preparation of tetrahydrobenzo[*b*]pyrans

Optimization of the reaction conditions for the synthesis of tetrahydrobenzo[*b*]pyrans was carried out in the presence of the Cu-BimG@SiO_2_@Fe_3_O_4_ catalyst. For this purpose, the reaction between 4-chlorobenzaldehyde (1 mmol), malononitrile (1 mmol), and dimedone (1 mmol) was carried out under different temperature conditions with different amounts of catalyst in polar and non-polar solvents, and solvent-free conditions. It was found that the reaction does not occur in the presence of non-polar solvents (Table 2, entries 2 and 3) and is possible in dichloromethane with a very low partial polarity (Table 2, entry 4). Furthermore, in solvent-free conditions, the reaction was tested at ambient temperature and 80 °C, but satisfactory results were not obtained; at ambient temperature, the reaction did not progress at all, and at 80 °C a partial product was formed (Table 2, entries 1 and 5). Next, the effects of polar solvents on the reaction were investigated. For this purpose, the reaction was carried out in water and ethanol as solvents. The results showed that the reaction with ethanol required a longer time (Table 2, entry 6), but the reaction in water showed satisfactory results (Table 2, entry 7). For this reason, to complete the optimization process, we investigated the effects of two factors, temperature and the amount of catalyst, in water as the solvent. The results showed that at ambient temperature in water, the reaction required a longer time than at 80 °C. This indicates the importance of temperature for the reaction. Also, with different amounts of catalyst, the reaction time varied slightly. Finally, the best conditions for the synthesis of tetrahydrobenzo[*b*]pyran derivatives in the presence of Cu-BimG@SiO_2_@Fe_3_O_4_ catalyst are water as the solvent at 80 °C in the presence of 15 mg of catalyst (Table 2, entry 9).

**Table 2:** Optimization of preparation conditions for tetrahydrobenzo[*b*]pyran derivatives in the presence of Cu-BimG@SiO_2_@Fe_3_O_4_ nanocatalyst

Entry	Solvent	Catalyst (mg)	Temperature (°C)	Time (min)	Yield (%)
1	—	20	80	120	Trace
2	Toluene	20	80	120	Trace
3	*n*-Hexane	20	70	60	N.R
4	Dichloromethane	20	r.t	120	N.R
5	—	20	r.t	60	N.R
6	Ethanol	20	80	330	86
7	H_2_O	20	80	15	97
8	H_2_O	20	r.t	105	48
**9**	**H**_**2**_ **O**	**15**	**80**	**15**	**97**
10	H_2_O	10	80	20	90

The synthesis of tetrahydrobenzo[*b*]pyrans was investigated in 2021 by Taherkhani *et al.* without any catalyst, and the corresponding products were not obtained even after long reaction times.^75^ The synthesis of tetrahydrobenzo[*b*]pyran derivatives was investigated in 2023 by Partovi *et al.* in the presence of Fe_3_O_4_ and SiO_2_@Fe_3_O_4_ nanoparticles as catalysts, and the corresponding products were obtained with yields of only 50% and 40%, respectively.^2^ Therefore, Fe_3_O_4_ and SiO_2_@Fe_3_O_4_ are not suitable catalysts for the synthesis of tetrahydrobenzo[*b*]pyrans. In this work, the synthesis of tetrahydrobenzo[*b*]pyrans was investigated in the presence of BimG@SiO_2_@Fe_3_O_4_ as a catalyst, and no significant product was obtained. Therefore, the main catalyst in the synthesis of tetrahydrobenzo[*b*]pyrans is Cu-BimG@SiO_2_@Fe_3_O_4_.

Next, in order to synthesize various tetrahydrobenzo[*b*]pyran derivatives, this method was carried out under optimal conditions using several aldehydes with different electron-withdrawing and electron-donating groups (Table 3). The results showed that the reaction with aldehydes bearing electron-withdrawing groups, such as nitro substituents, takes place in a shorter time than with other aldehydes. One reason that can be mentioned for this is that these groups accelerate the Knoevenagel condensation because they activate the carbonyl of the aldehyde and therefore facilitate the nucleophilic attack. Finally, it can be claimed that the results of the tetrahydrobenzo[*b*]pyran synthesis reaction in the presence of the Cu-BimG@SiO_2_@Fe_3_O_4_ catalyst were satisfactory for all derivatives.

**Table 3:** Synthesis of tetrahydrobenzo[*b*]pyran derivatives in the presence of Cu-BimG@SiO_2_@Fe_3_O_4_ nanocatalyst

Entry	Aldehyde	Product	Time (min)	Yield (%)
1	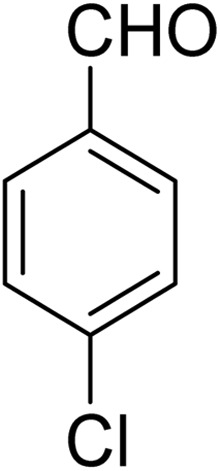	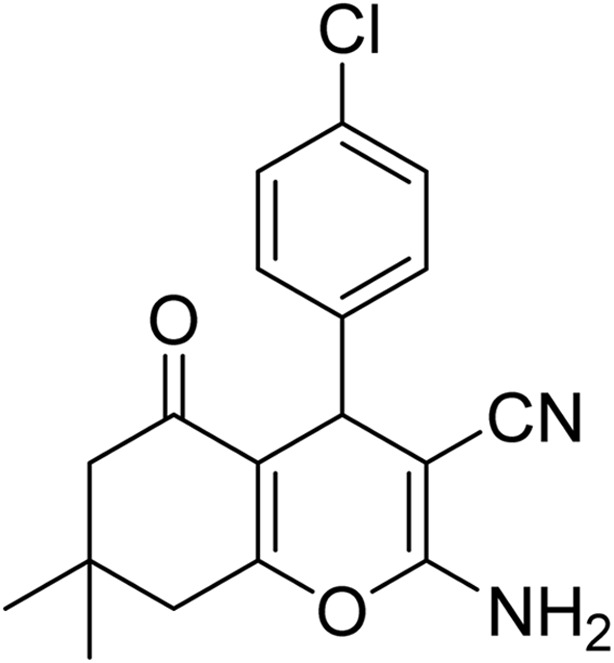	15	97
2	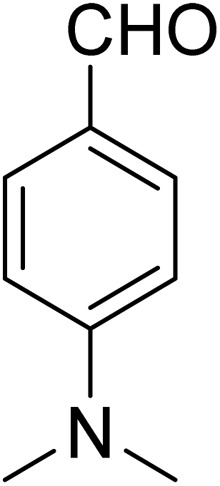	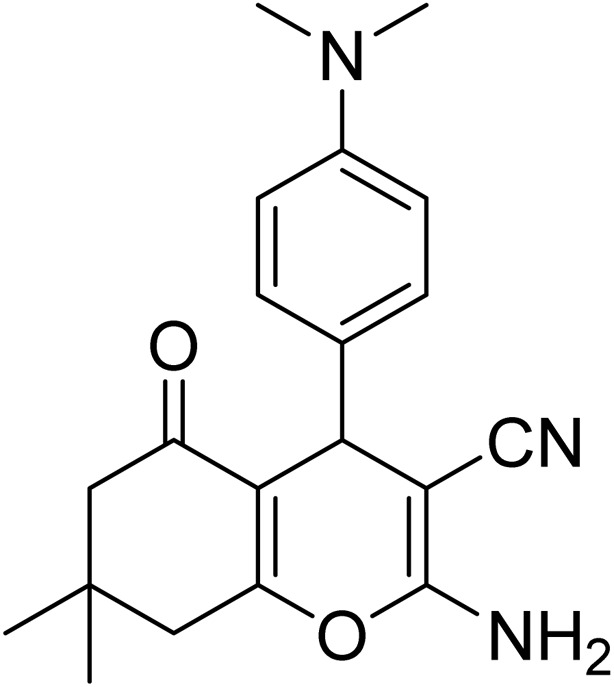	120	90
3	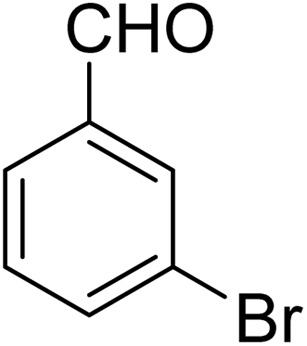	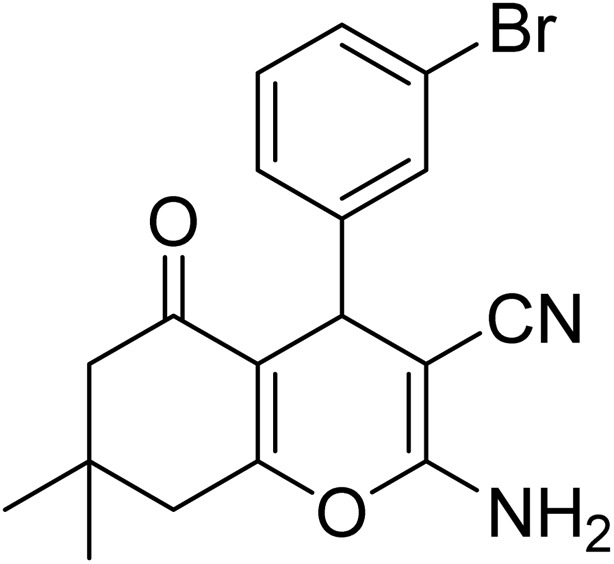	20	89
4	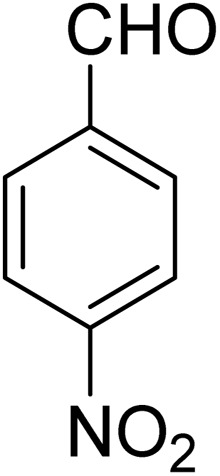	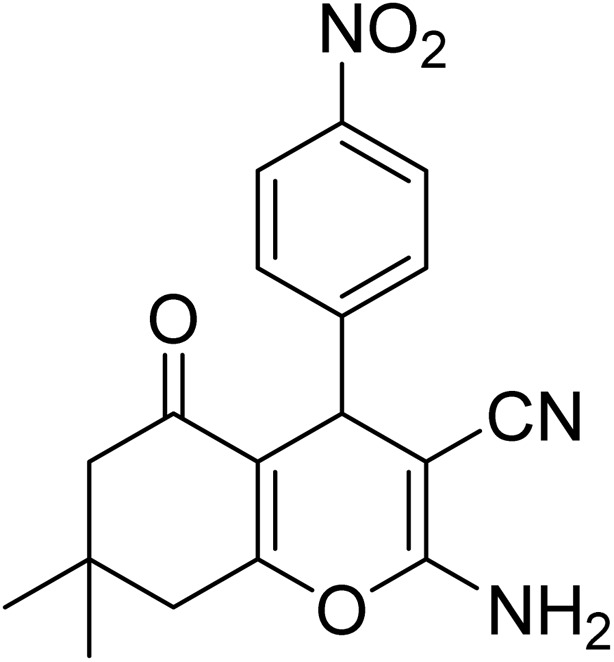	10	83
5	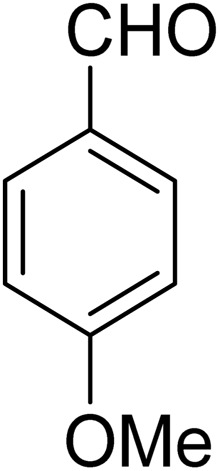	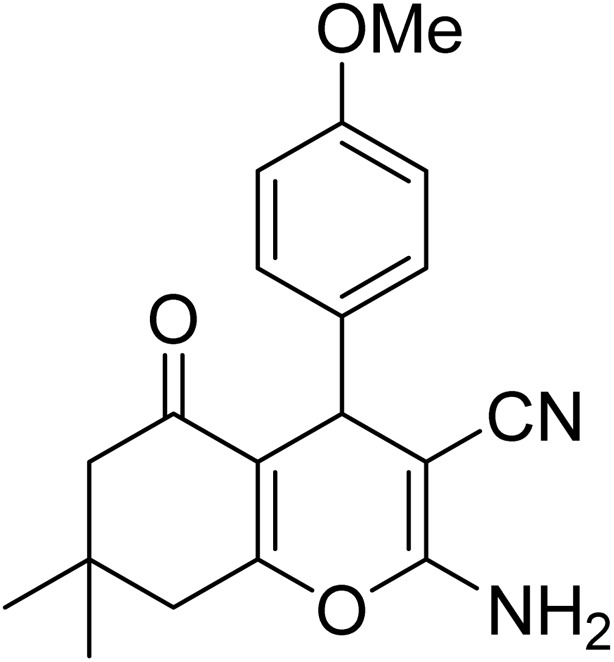	20	98
6	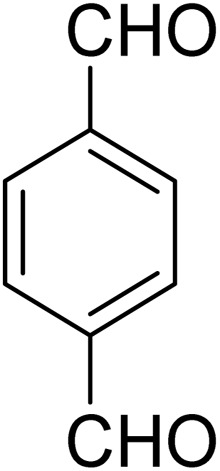	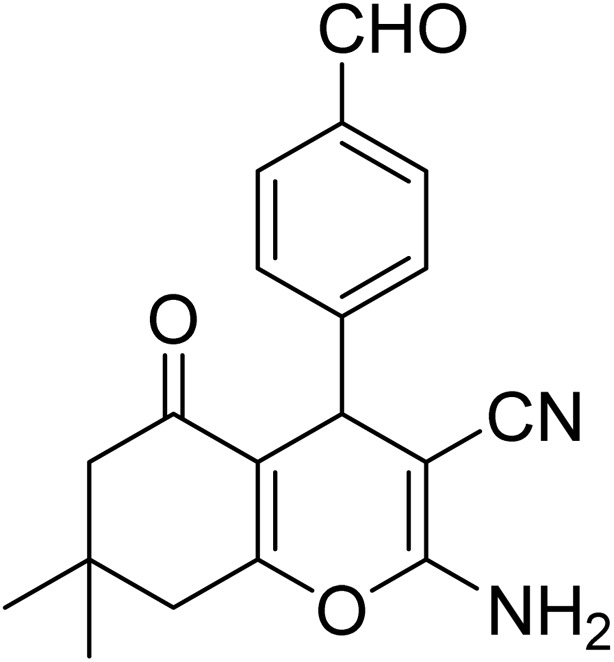	40	80
7	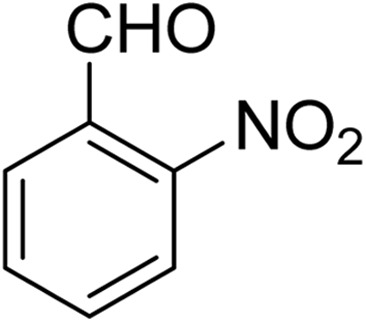	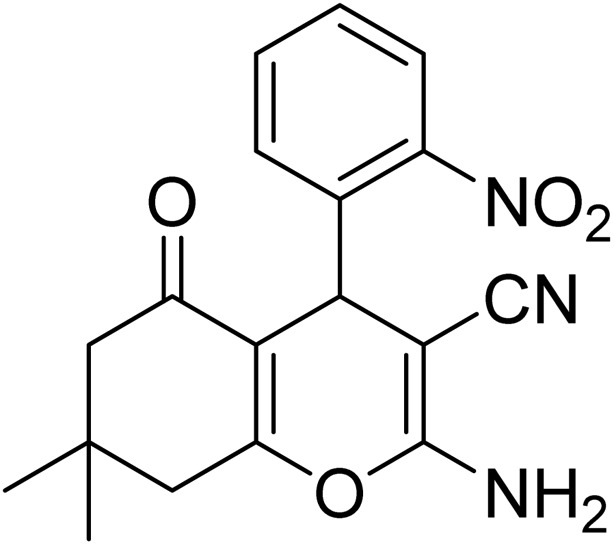	10	90
8	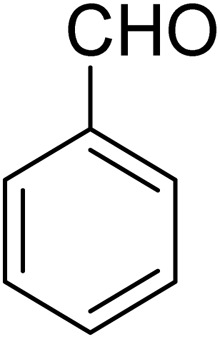	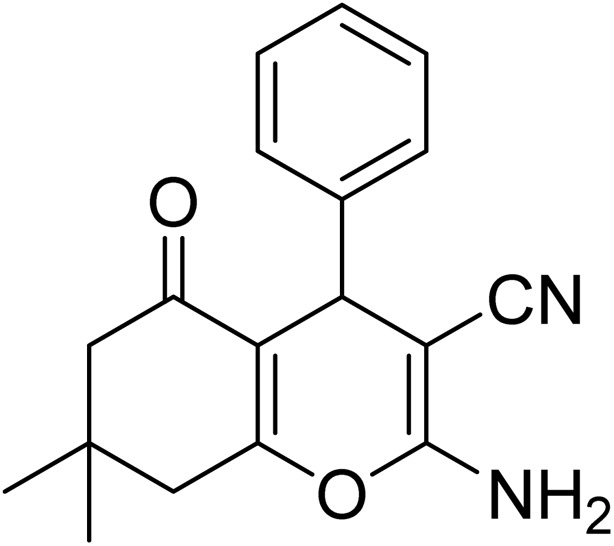	12	63
9	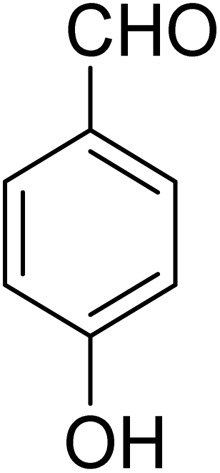	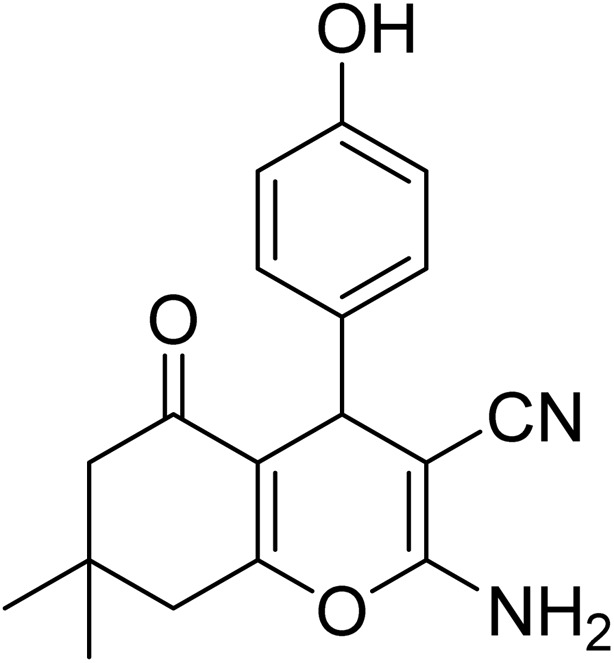	12	76
10	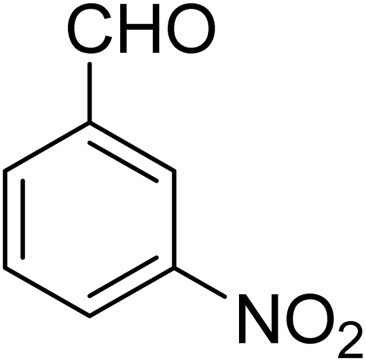	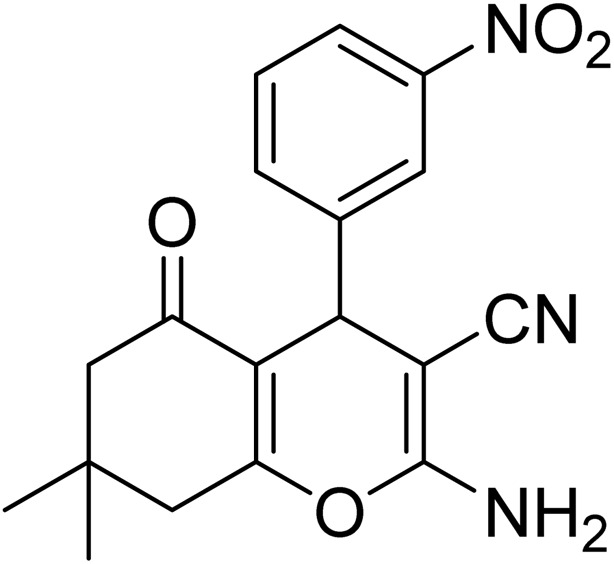	7	89
11	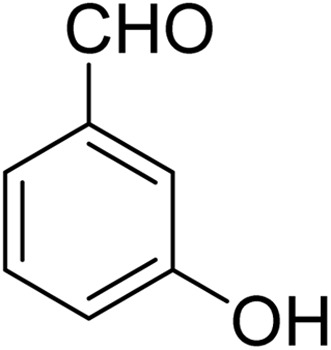	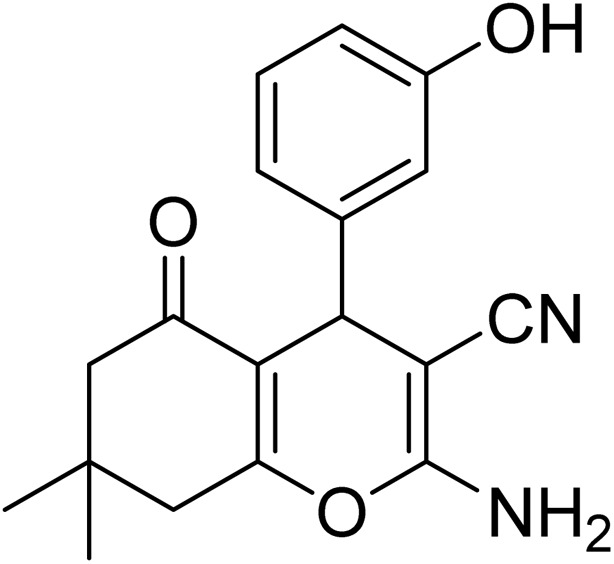	15	83

**Scheme 5:**
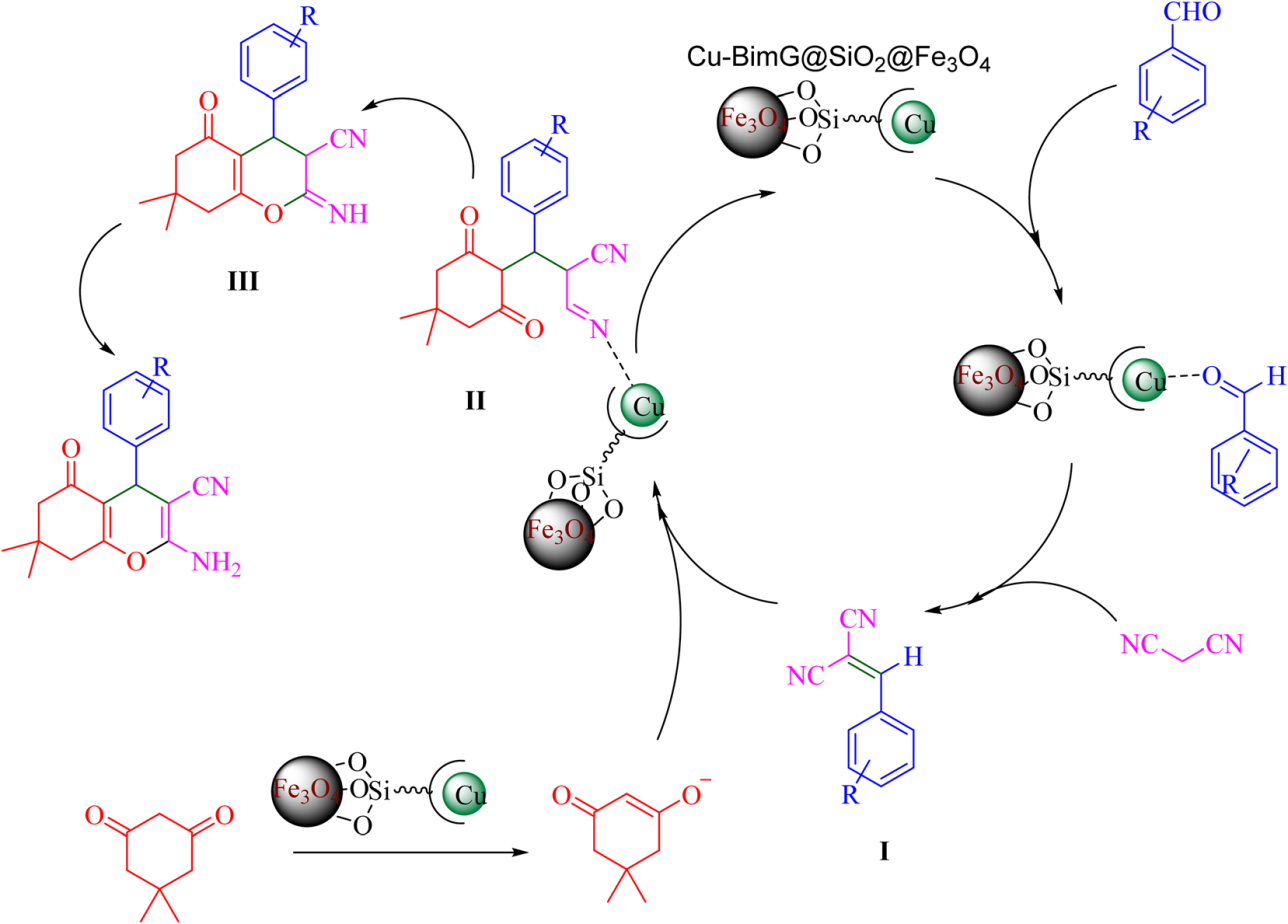
Proposed mechanism for the synthesis of tetrahydrobenzo[*b*]pyran catalyzed by Cu-BimG@SiO_2_@Fe_3_O_4_.

Our proposed mechanism for the synthesis of tetrahydrobenzo[*b*]pyran derivatives in the presence of Cu-BimG@SiO_2_@Fe_3_O_4_ catalyst, according to previous references (Scheme 5),^80^ involves the first step of performing the Knoevenagel condensation between malononitrile and aldehyde, followed by a Michael addition reaction between dimedone and the product of the first step, and finally producing the desired tetrahydrobenzo[*b*]pyran product.

#### Homoselectivity study of Cu-BimG@SiO_2_@Fe_3_O_4_

**Scheme 6:**
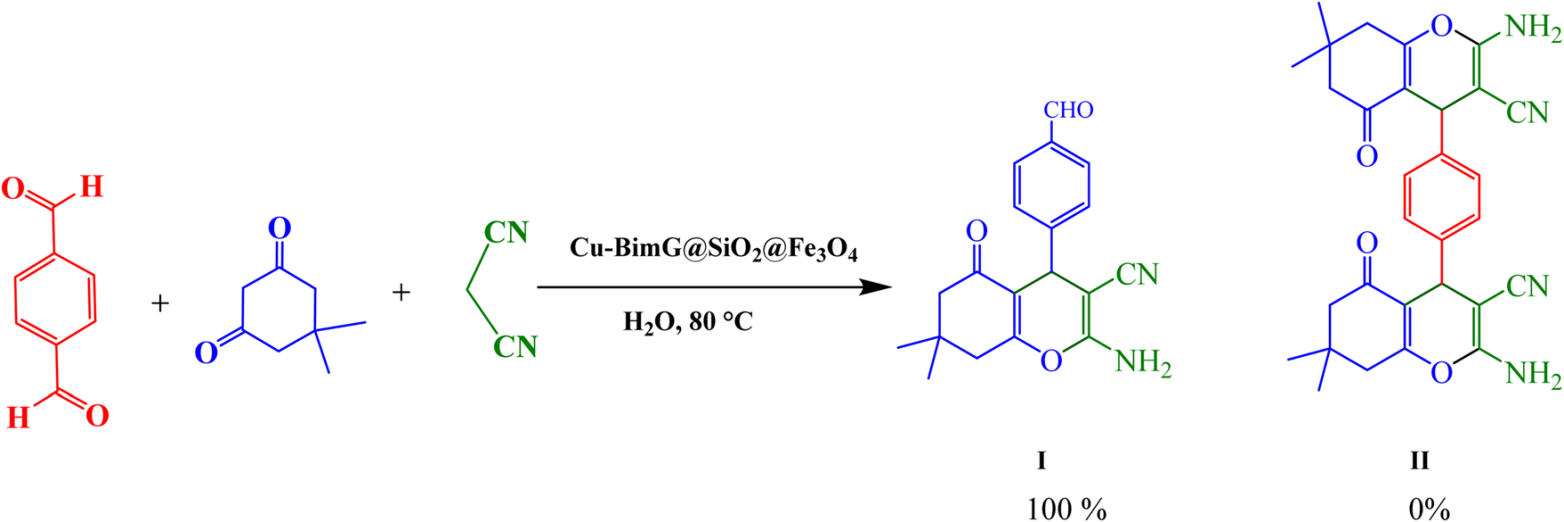
Homoselectivity of Cu-BimG@SiO_2_@Fe_3_O_4_ in the condensation of terephthalaldehyde with dimedone and malononitrile.

**Fig. 9:**
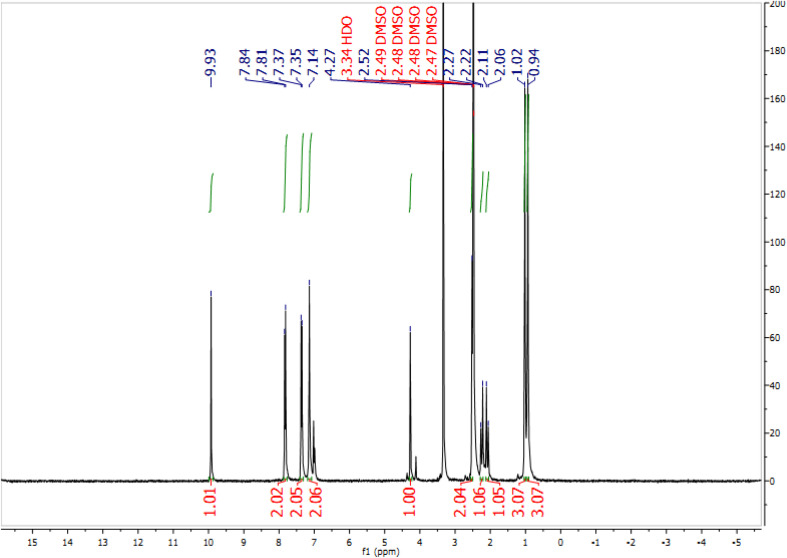
^1^H NMR spectrum of 2-amino-4-(4-formylphenyl)-7,7-dimethyl-5-oxo-5,6,7,8-tetrahydro-4*H*-chromene-3-carbonitrile.

Cu-BimG@SiO_2_@Fe_3_O_4_ provides good homoselectivity in the formation of tetrahydrobenzo[*b*]pyrans. As shown, in the three-condensation of dimedone, malononitrile and terephthalaldehyde, which have two quite similar carbaldehyde substrate groups in their structures, only mono-condensation was observed in the presence of the Cu-BimG@SiO_2_@Fe_3_O_4_ catalyst (Scheme 6). The amount of Cu-BimG@SiO_2_@Fe_3_O_4_ in the condensation of dimedone, malononitrile, and terephthalaldehyde toward the synthesis of (I) or (II) was investigated using ^1^H NMR spectroscopy. As mentioned, one of the carbaldehyde functional groups selectively participates in the reaction for the synthesis of the corresponding product, and the other functional group remains unchanged. This remaining carbaldehyde functional group is characterized by a single peak at around 9.93 ppm in the ^1^H NMR spectrum. However, if both carbaldehyde groups participate in the reaction, this peak does not appear in the ^1^H NMR spectrum. Therefore, only product I is homoselectively formed in the presence of Cu-BimG@SiO_2_@Fe_3_O_4_ (Fig. 9). Furthermore, in the ^1^H NMR spectrum of I, two peaks for aromatic hydrogens should be observed, while in the ^1^H NMR spectrum of II, one peak for aromatic hydrogens should be observed. As can be seen in the ^1^H NMR spectrum of the final synthesized product, two peaks were observed at 7.84–7.81 ppm and 7.31–7.35 ppm, as follows: ^1^H NMR (300 MHz, DMSO_d6_): *δ*_H_ = 9.93 (s, 1H), 7.84–7.81 (d, *J* = 9 Hz, 2H), 7.37–7.35 (d, *J* = 6 Hz, 2H), 7.14 (br, 2H), 4.27 (s, 1H), 2.52 (s, 2H), 2.27–2.22 (d, *J* = 15 Hz, 1H), 2.11–2.06 (d, *J* = 15 Hz, 1H), 1.02 (s, 3H), 0.94 (s, 3H) ppm. Thus, product II is not formed. Therefore, based on the NMR data, only 2-amino-4-(4-formylphenyl)-7,7-dimethyl-5-oxo-5,6,7,8-tetrahydro-4*H*-chromene-3-carbonitrile is selectively formed in the presence of the Cu-BimG@SiO_2_@Fe_3_O_4_ catalyst.

Due to the diastereotopic nature of CH_2_'s hydrogen, they appeared as a doublet-of-doublet peak in the ^1^H NMR spectrum in the region of 2.27–2.22 ppm and 2.11–2.06 ppm.

#### Reusability and Cu leaching study of Cu-BimG@SiO_2_@Fe_3_O_4_

**Fig. 10:**
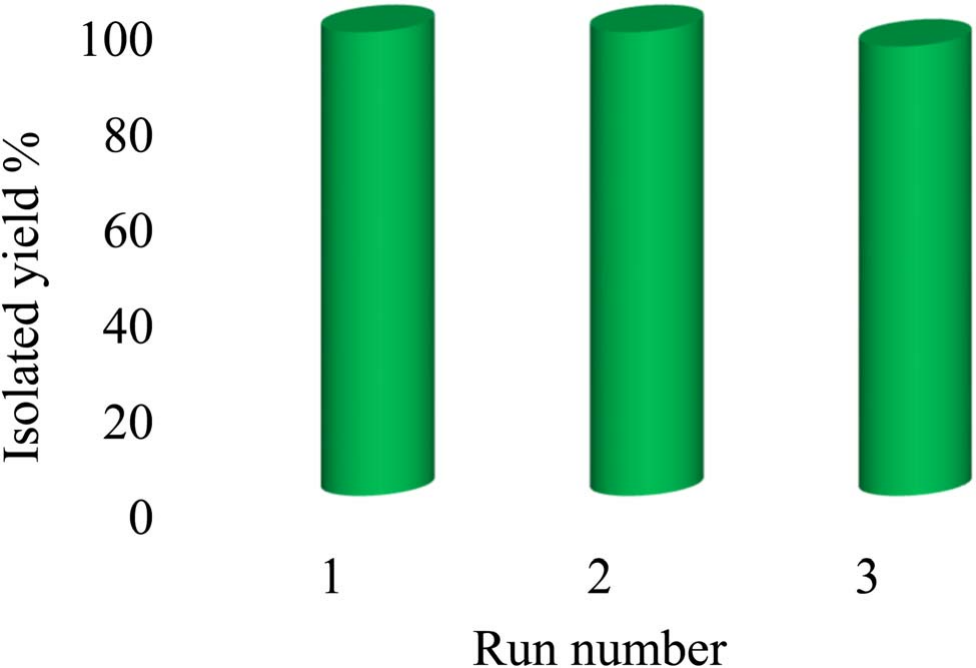
Reuse of the Cu-BimG@SiO_2_@Fe_3_O_4_ catalyst for the preparation of tetrahydrobenzo[*b*]pyran.

**Fig. 11:**
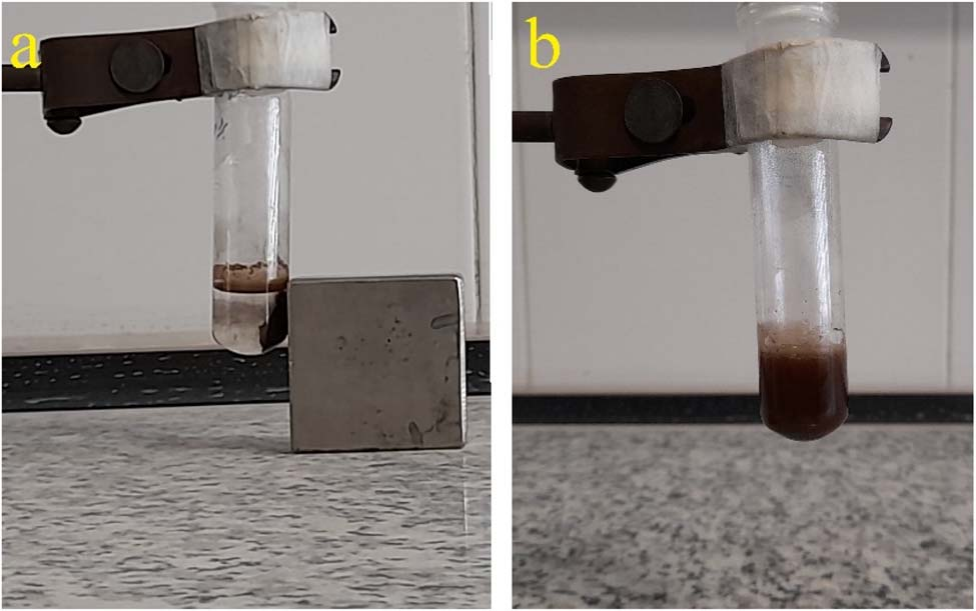
Magnetic separation of Cu-BimG@SiO_2_@Fe_3_O_4_ catalyst: (a) the reaction mixture in the presence of an external magnet, and (b) the reaction mixture in the absence of an external magnet.

The ability to be recycled and reused is one of the attractive features of heterogeneous catalysts. In order to investigate this feature for the Cu-BimG@SiO_2_@Fe_3_O_4_ catalyst in the synthesis tetrahydrobenzo[*b*]pyran derivatives, we designed a model reaction based on optimal conditions using the multicomponent reaction of 4-chlorobenzaldehyde, malononitrile, and dimedone. As we predicted, the aforementioned catalyst provided good performance in terms of reaction time and efficiency after three cycles of recycling and reuse (Fig. 10). In this study, the catalyst was recovered after each step using an external magnet (Fig. 11) and washed. The recovered catalyst was then reused in the next step under the same conditions. This process was repeated up to three times.

The heterogeneous nature of the Cu-BimG@SiO_2_@Fe_3_O_4_ catalyst was studied by a hot filtration test and AAS analysis. For this investigation, the synthesis of 2-amino-4-(4-chlorophenyl)-7,7-dimethyl-5-oxo-5,6,7,8-tetrahydro-4*H*-chromene-3-carbonitrile from the multicomponent reaction of 4-chlorobenzaldehyde, malononitrile, and dimedone was selected under optimized conditions in Table 2. The Cu-BimG@SiO_2_@Fe_3_O_4_ catalyst was removed by magnetic decantation after 15 min, and the remaining solution was analyzed using the AAS technique. No notable Cu leaching was observed in the solution (exact amount of Cu leached was determined to be 0.00028 mol L^−1^). Therefore, it can be said with certainty that copper leaching from the catalyst did not occur in solution under reaction conditions.

#### Characterization of the recovered Cu-BimG@SiO_2_@Fe_3_O_4_ catalyst

The recovered Cu-BimG@SiO_2_@Fe_3_O_4_ nanocatalyst was characterized by SEM, EDS, WDX, and FTIR, and was compared with fresh Cu-BimG@SiO_2_@Fe_3_O_4_ catalyst.

**Fig. 12:**
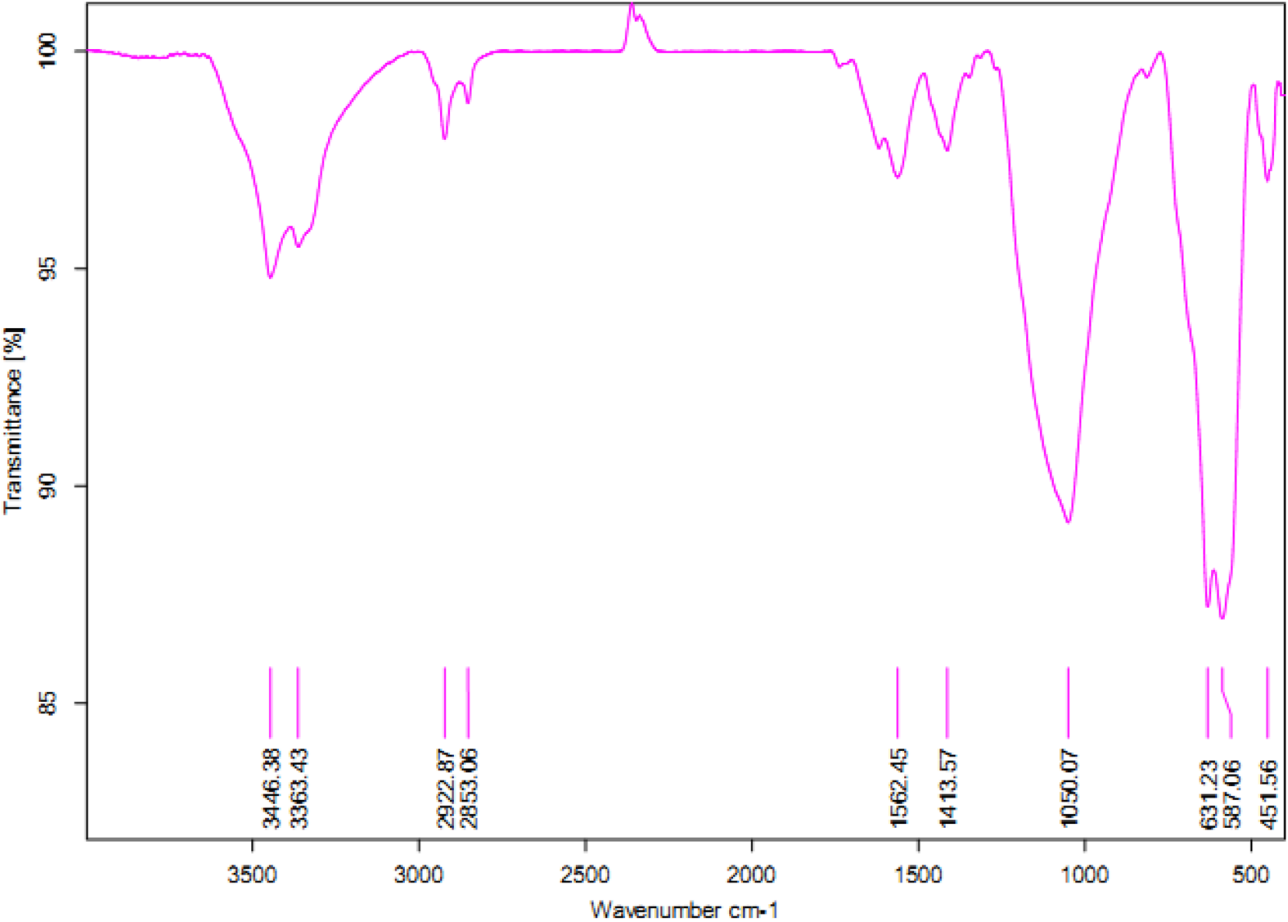
FTIR spectrum of the recovered Cu-BimG@SiO_2_@Fe_3_O_4_ nanocatalyst.

Fig. 12 illustrates the FTIR spectrum of the recovered Cu-BimG@SiO_2_@Fe_3_O_4_ catalysts. The FTIR spectrum of the recovered catalyst is in perfect agreement with the FTIR spectrum of the fresh catalyst.

**Fig. 13:**
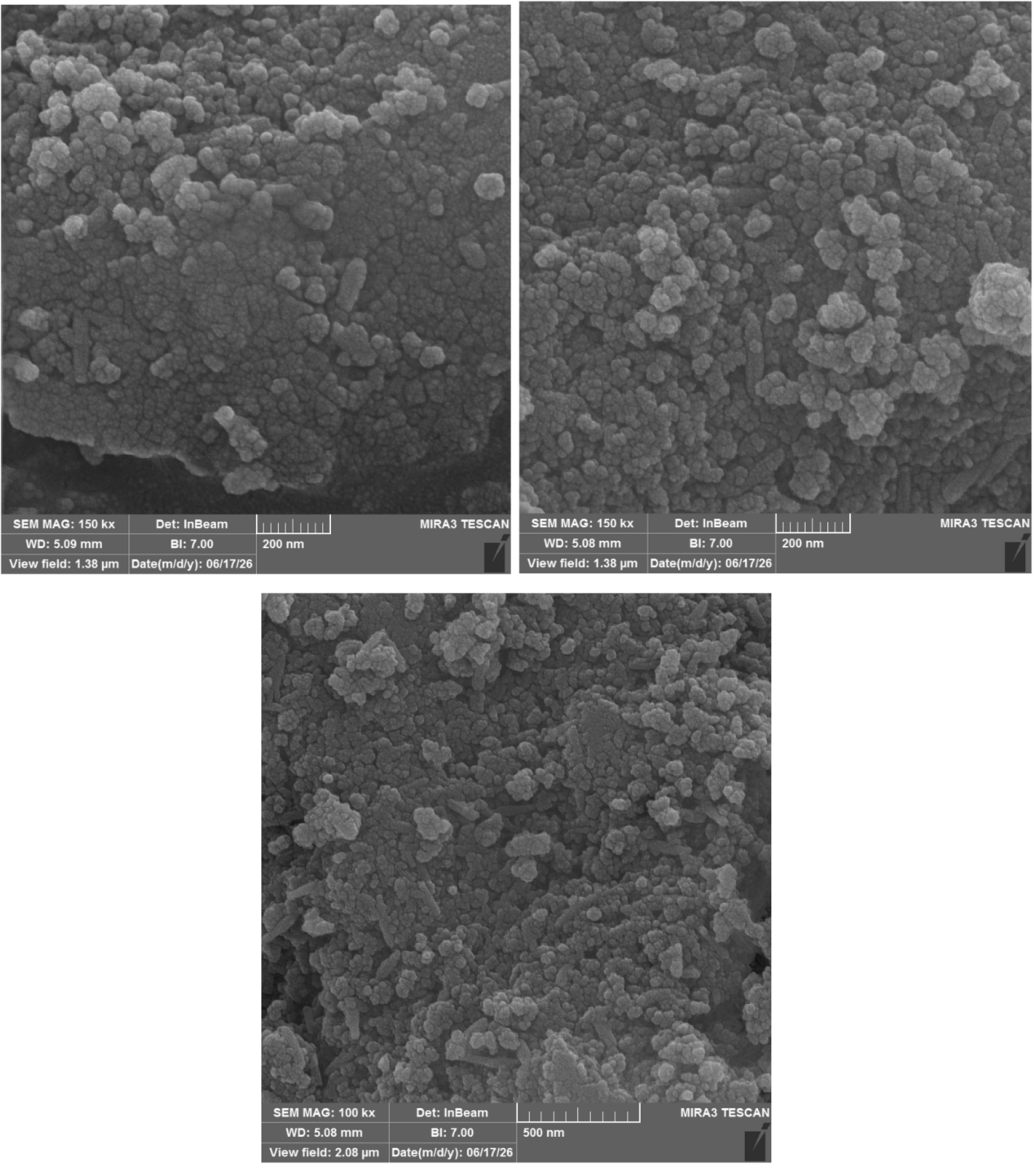
SEM images of the recovered Cu-BimG@SiO_2_@Fe_3_O_4_ nanocatalyst.

Fig. 13 illustrates the SEM images of the recovered Cu-BimG@SiO_2_@Fe_3_O_4_ catalyst. These images show that this catalyst has a uniform particle size and morphology, similar to that of the fresh Cu-BimG@SiO_2_@Fe_3_O_4_ catalyst. The morphology and particle size of the catalyst remained unchanged after recycling.

**Fig. 14:**
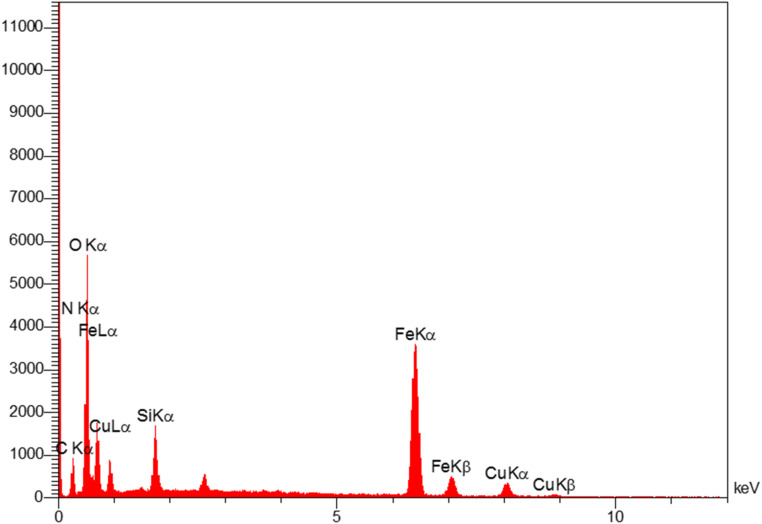
EDS analysis of the recovered Cu-BimG@SiO_2_@Fe_3_O_4_ nanocatalyst.

**Fig. 15:**
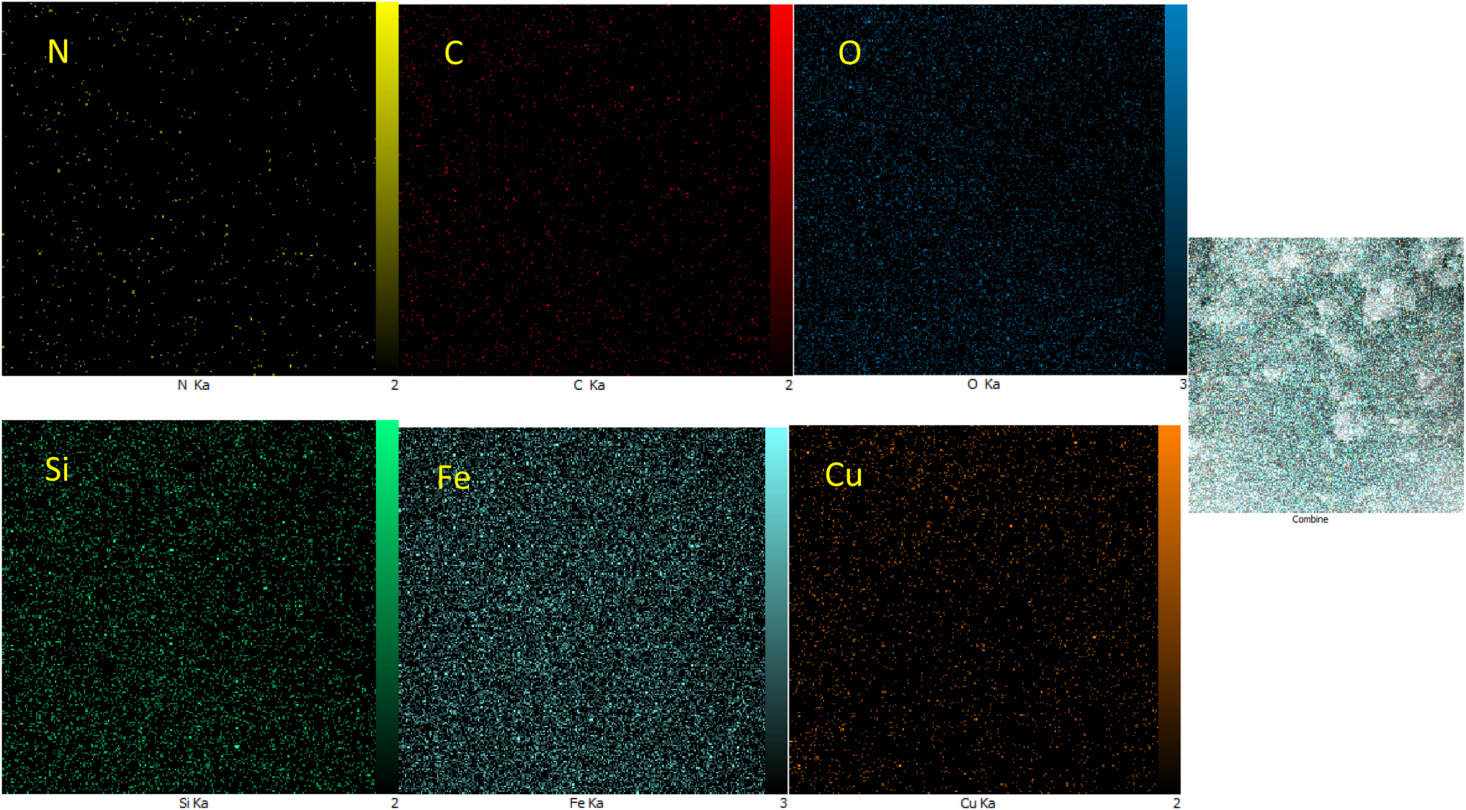
WDX elemental mapping images of the recovered Cu-BimG@SiO_2_@Fe_3_O_4_ nanocatalyst.

The EDS spectrum and WDX mapping of the recovered Cu-BimG@SiO_2_@Fe_3_O_4_ catalyst are shown in Fig. 14 and 15. The EDS results for the recovered catalyst (Fig. 14) confirm the presence of the C, N, O, Si, Fe, and Cu species, similar to the fresh catalyst. Furthermore, the WDX results for the recovered catalyst (Fig. 15) show that C, N, O, Si, Fe, and Cu elements are uniformly dispersed in the structure of the recovered nanocatalyst.

### Comparison of the catalyst

The performance of the Cu-BimG@SiO_2_@Fe_3_O_4_ catalyst was compared with other reported catalysts for the preparation of tetrahydrobenzo[*b*]pyran derivatives in terms of amount of catalyst, solvent, temperature, recyclability, reaction time, and yield. All reactions in Table 4 were carried out using 1 mmol of starting material, except for entry 7, which is reported on a 0.125 mmol scale. The reaction between 4-methoxybenzaldehyde, malononitrile, and dimedone was selected for this comparison (Table 4). It was observed that the Cu-BimG@SiO_2_@Fe_3_O_4_ catalyst gave better results in terms of reaction time and efficiency. The Cu-BimG@SiO_2_@Fe_3_O_4_ catalyst can be recovered and reused several times. The selective synthesis of tetrahydrobenzo[*b*]pyran derivatives in the presence of the Cu-BimG@SiO_2_@Fe_3_O_4_ catalyst has been investigated, while the catalyst selectivity has not been investigated for any of the mentioned catalysts in Table 4.

**Table 4:** Comparison of the efficiency of Cu-BimG@SiO_2_@Fe_3_O_4_ with catalysts reported in the literature for the synthesis of tetrahydrobenzo[*b*]pyran derivatives

Entry	Catalyst	Reaction conditions	Time (min)	Yield (%)	Ref.
1	Mesalamine/GPTMS@SiO_2_@Fe_3_O_4_ (0.07 g)	Dry grinding, r.t.	35	89	2
2	Fe_3_O_4_@SiO_2_@GPTMS@guanidine (0.02 g)	H_2_O, r.t.	360	92	75
3	LAMNP (0.04 g)	H_2_O/EtOH, 70 °C	30	90	81
4	POPINO (5 mol%)	H_2_O, reflux	25	89	82
5	NFDSiPD (0.02 g)	Solvent-free, 60 °C	30	84	83
6	WEMFSA (5 mL)	EtOH, r.t.	60	87	84
7	Fe_3_O_4_@NFC/E-CHDA-Cu^II^ (1 mol% per 0.1 mmol)	Solvent-free, r.t.	80	87	85
8	Cu-BimG@SiO_2_@Fe_3_O_4_ (0.015 g)	H_2_O, 80 °C	20	98	This work

## Conclusion

In this study, a Cu-BimG@SiO_2_@Fe_3_O_4_ nanocatalyst with good performance was used in the synthesis of tetrahydrobenzo[*b*]pyran derivatives, and satisfactory results were obtained. In order to confirm the structure of the catalyst, TGA, BET, FTIR, WDX, EDS, XRD, SEM, AAS, and VSM techniques were used. This catalyst can be easily recovered by an external magnet, and the mentioned catalyst showed good recyclability and reusability. The Cu-BimG@SiO_2_@Fe_3_O_4_ nanocatalyst showed good selectivity in the synthesis of tetrahydrobenzo[*b*]pyran derivatives, which was confirmed by NMR spectroscopy.

## Conflicts of interest

There are no conflicts to declare.

## Supplementary Material

RA-OLF-D6RA02796B-s001

## Data Availability

All data generated or analyzed during this study are included in this published article and its supplementary information (SI). Supplementary information is available. See DOI: https://doi.org/10.1039/d6ra02796b.
