# Supplementary material for: A new copper complex of 1-(1H-benzo[d]imidazol-2-yl)guanidine on magnetic Fe3O4 nanoparticles as a green, reusable, robust and homoselective nanocatalyst in the synthesis of tetrahydrobenzo[b]pyrans
Source: RSC Adv. 2026 Jul 21;16(38):41913–30. doi: 10.1039/d6ra02796b (PMC13384768; doi:10.1039/d6ra02796b)

Supplementary Information (SI) for RSC Advances.
This journal is © The Royal Society of Chemistry 2026

Supplementary Data

A new copper complex of 1-(1H-benzo[d]imidazol-2-yl)guanidine on magnetic Fe_3O_4 nanoparticles as a green, returnable, robust and homoselective nanocatalyst in the synthesis of tetrahydrobenzo[b]pyrans

Bahman Tahmasbi*, Abdolkhalegh Hosien Ali, Saeid Taghavi Fardood, Omid Soleimani

Department of Chemistry, Faculty of Science, Ilam University, P. O. Box69315516, Ilam, Iran. E-mail address: b.tahmasbi@ilam.ac.ir

Abstract

In this work, the magnetic Fe_3O_4 nanoparticles were encapsulated using a silica layer via a sol-gel method, which was labeled as SiO_2@Fe_3O_4. Then, the surface of SiO_2@Fe_3O_4 was modified using 3-iodopropyltrimethoxysilane (3-IPTMS), denoted as IPTMS@SiO_2@Fe_3O_4. Also, 1-(1H-benzo[d]imidazol-2-yl)guanidine ligand (BimG) was synthesized from the condensation of cyanoguanidine and benzene-1,2-diamine in acidic conditions. Then, a new copper complex of BimG was anchored on the surface of IPTMS@SiO_2@Fe_3O_4, which was labeled as Cu-BimG@SiO_2@Fe_3O_4. This nanocatalyst was identified with TGA, BET, WDX, EDS, FTIR, XRD, SEM, AAS, and VSM techniques. Cu-BimG@SiO_2@Fe_3O_4 was investigated as an effective, highly homoselective and returnable nanocatalyst for the synthesizing of tetrahydrobenzo[b]pyrans through a multicomponent reaction. The homoselectivity of this catalyst was confirmed by NMR spectroscopy. This Cu-BimG@SiO_2@Fe_3O_4 catalyst showed good reusability for up to several runs without a meaningful loss of catalytic performance. The recovered Cu-BimG@SiO_2@Fe_3O_4 nanocatalyst was characterized by SEM, EDS, WDX, and FTIR techniques, which were matched with a fresh catalyst.

Keywords: Magnetic Fe_3O_4 nanoparticles, Encapsulated nanoparticles, Multi-component reactions, Copper, Tetrahydrobenzo[b]pyrans, Hemoselective nanocatalyst

*Address correspondence to Department of Chemistry, Faculty of Science, Ilam University, P. O. Box 69315516, Ilam, Iran. E-mail address of B. Tahmasbi: b.tahmasbi@ilam.ac.ir and bah.tahmasbi@gmail.com

¹H NMR spectral data

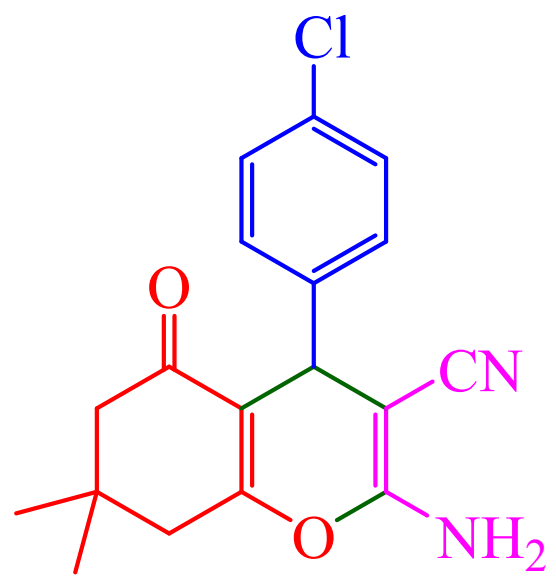

2-amino-4-(4-chlorophenyl)-7,7-dimethyl-5-oxo-5,6,7,8-tetrahydro-4H-chromene-3-carbonitrile

^{1}H NMR (250 MHz, $DMSO_{d6}$): δ_H= 7.35-7.32 (d, *J*= 7.5 Hz, 2H), 7.13-7.16 (d, *J*= 7.5 Hz, 2H), 7.06 (br, 2H), 4.17 (s, 1H), 2.49 (s, 2H), 2.27-2.20 (d, *J*= 17.5 Hz, 1H), 2.11-2.05 (d, *J*= 15 Hz, 1H), 1.01 (s, 3H), 0.93 (s, 3H) ppm.

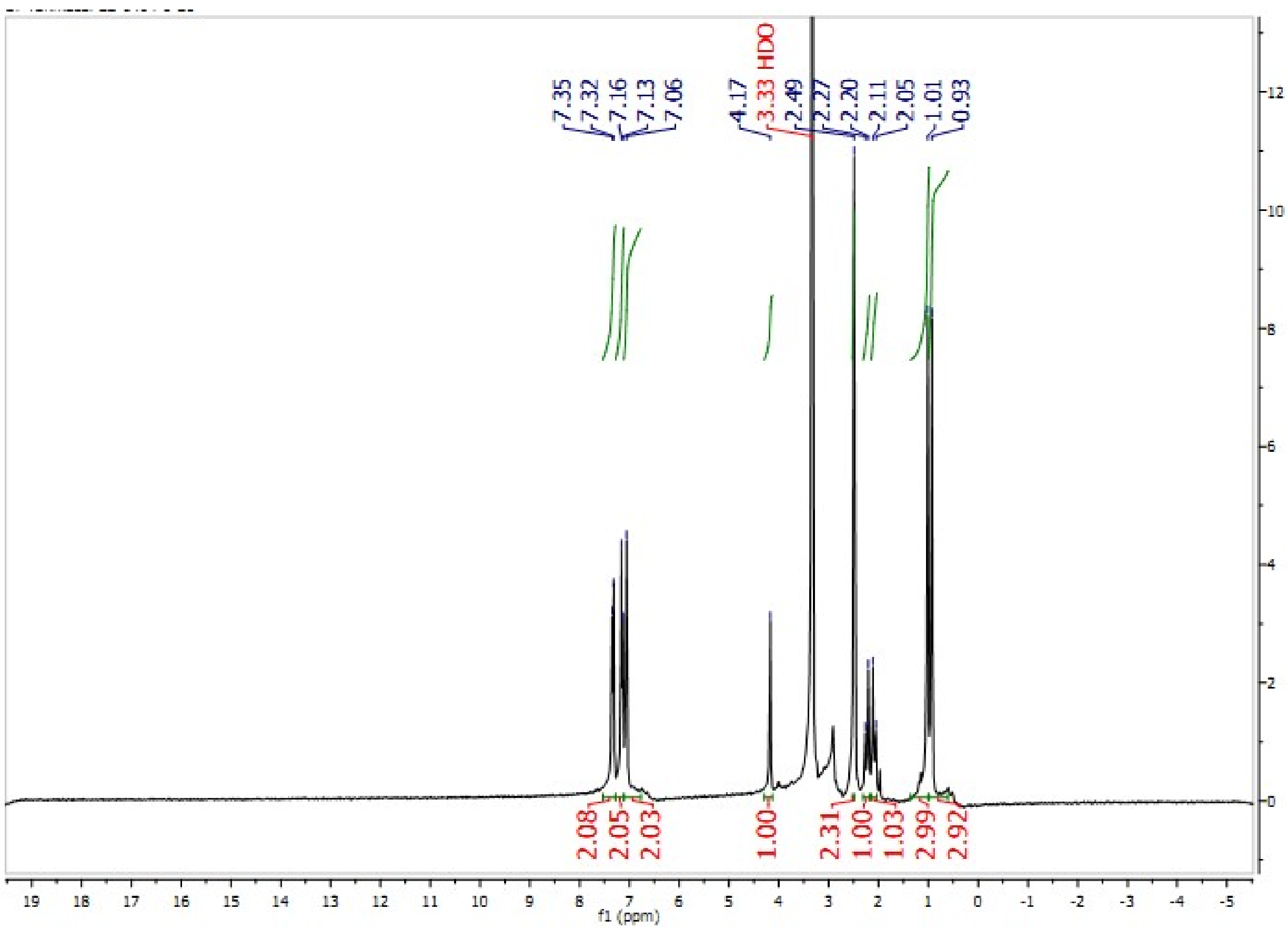

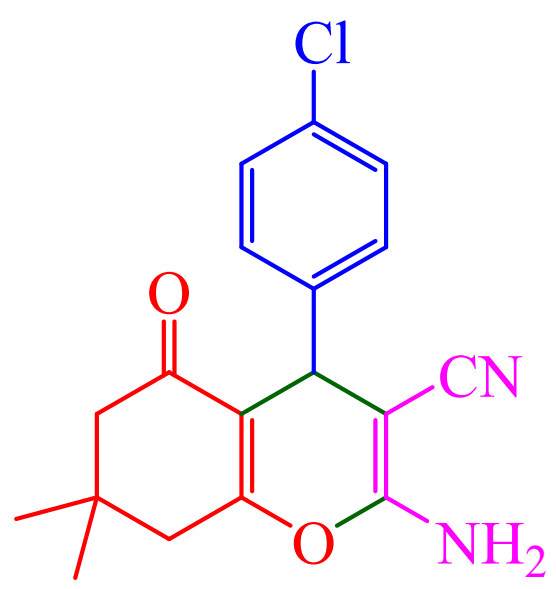

2-amino-4-(4-chlorophenyl)-7,7-dimethyl-5-oxo-5,6,7,8-tetrahydro-4H-chromene-3-carbonitrile

IR (KBr) cm^{-1}: 3380, 3182, 2958, 2889, 2188, 1677, 1634, 1604, 1490, 1412, 1365, 1309, 1286, 1247, 1216, 1162, 1139, 1093, 1032, 1014, 973, 939, 917, 853, 828, 769, 682, 619, 562, 520, 472.

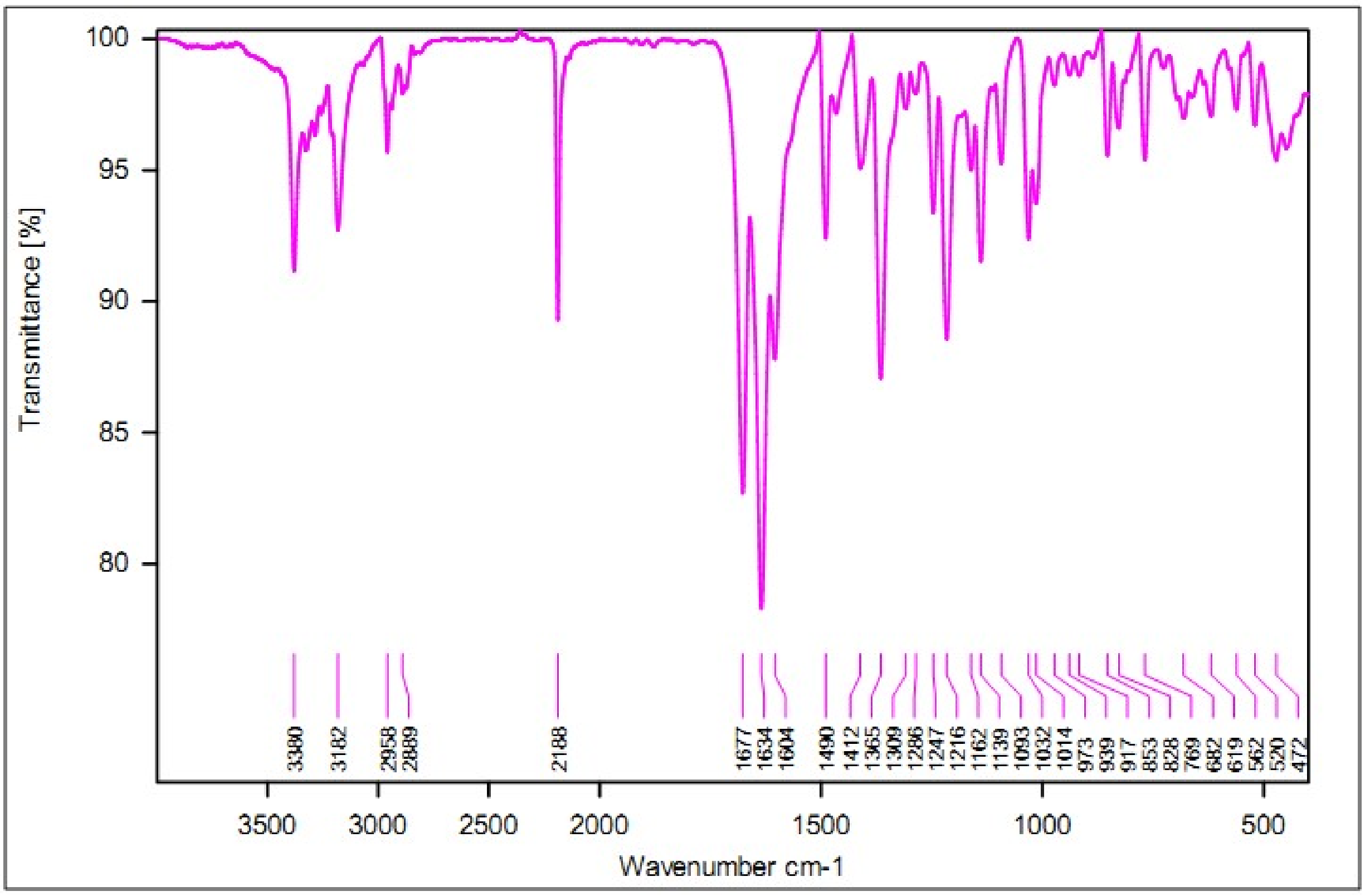

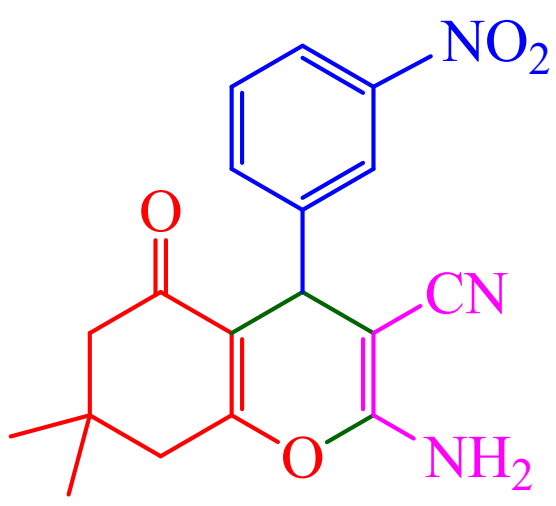

2-amino-7,7-dimethyl-4-(3-nitrophenyl)-5-oxo-5,6,7,8-tetrahydro-4H-chromene-3-carbonitrile

^{1}H NMR (250 MHz, DMSO$_{d6}$): δ_H= 7.80-7.78 (d, *J*= 5 Hz, 1H), 7.64 (s, 1H), 7.43-7.31 (m, 2H), 7.19 (br, 2H), 4.90 (s, 1H), 2.48 (s, 2H), 2.21-2.15 (d, *J*= 15 Hz, 1H), 2.02-1.95 (d, *J*= 17.5 Hz, 1H), 0.99 (s, 3H), 0.85 (s, 3H) ppm.

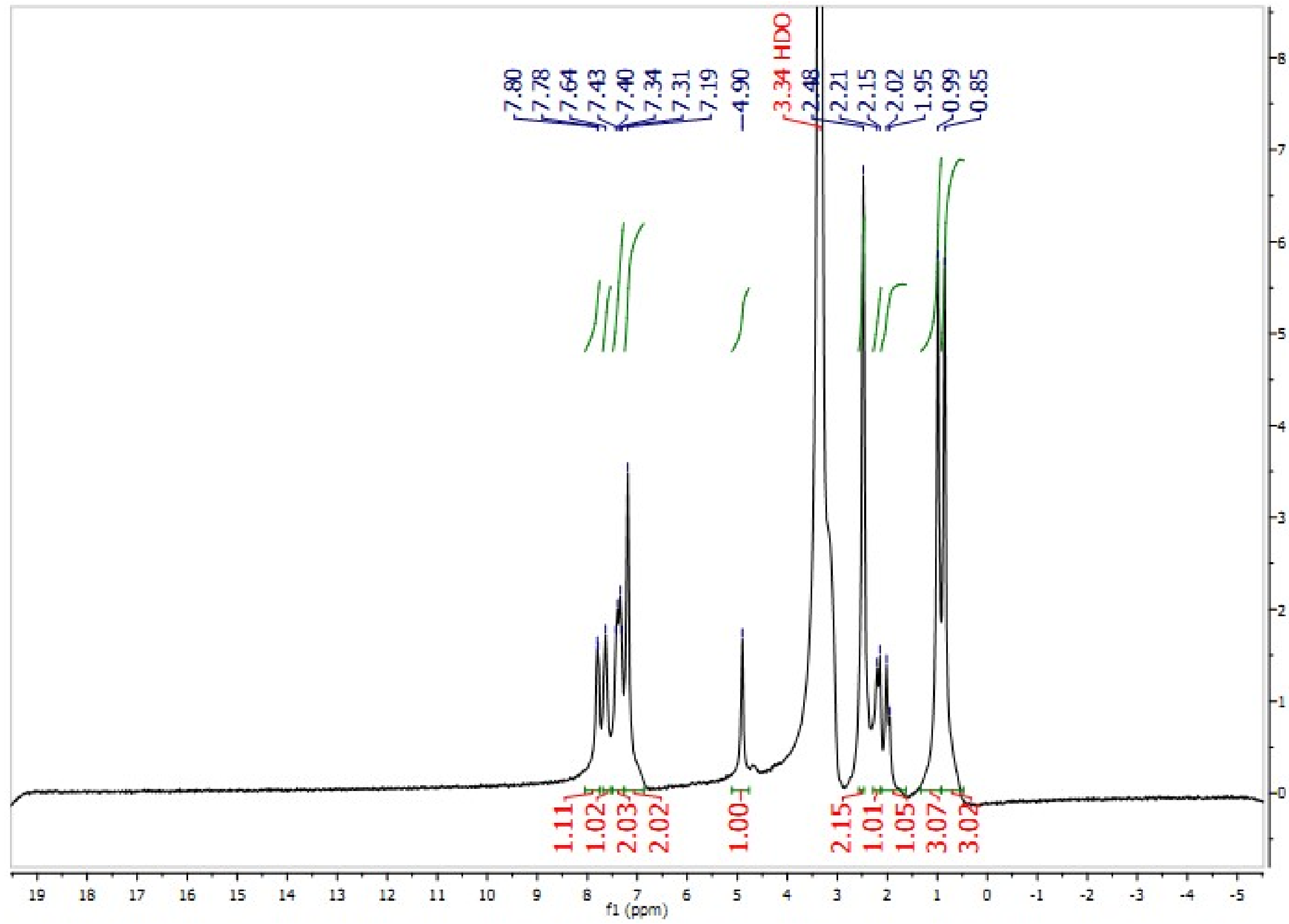

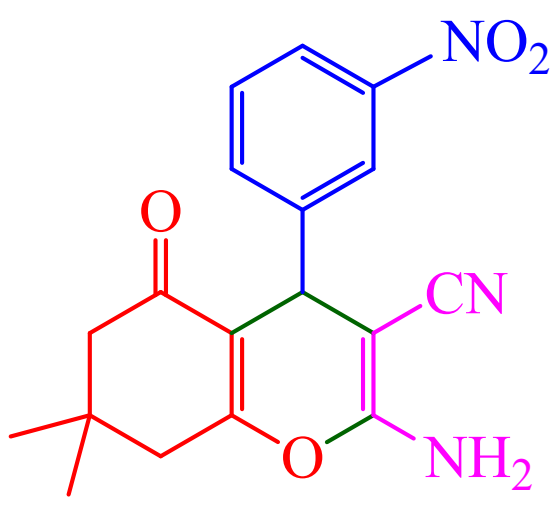

2-amino-7,7-dimethyl-4-(3-nitrophenyl)-5-oxo-5,6,7,8-tetrahydro-4H-chromene-3-carbonitrile

IR (KBr) cm^{-1}: 3476, 3332, 3253, 3206, 2960, 2870, 2836, 2198, 1686, 1662, 1597, 1526, 1468, 1414, 1362, 1254, 1214, 1143, 1041, 976, 917, 861, 826, 785, 737, 703, 677, 644, 562, 518, 434.

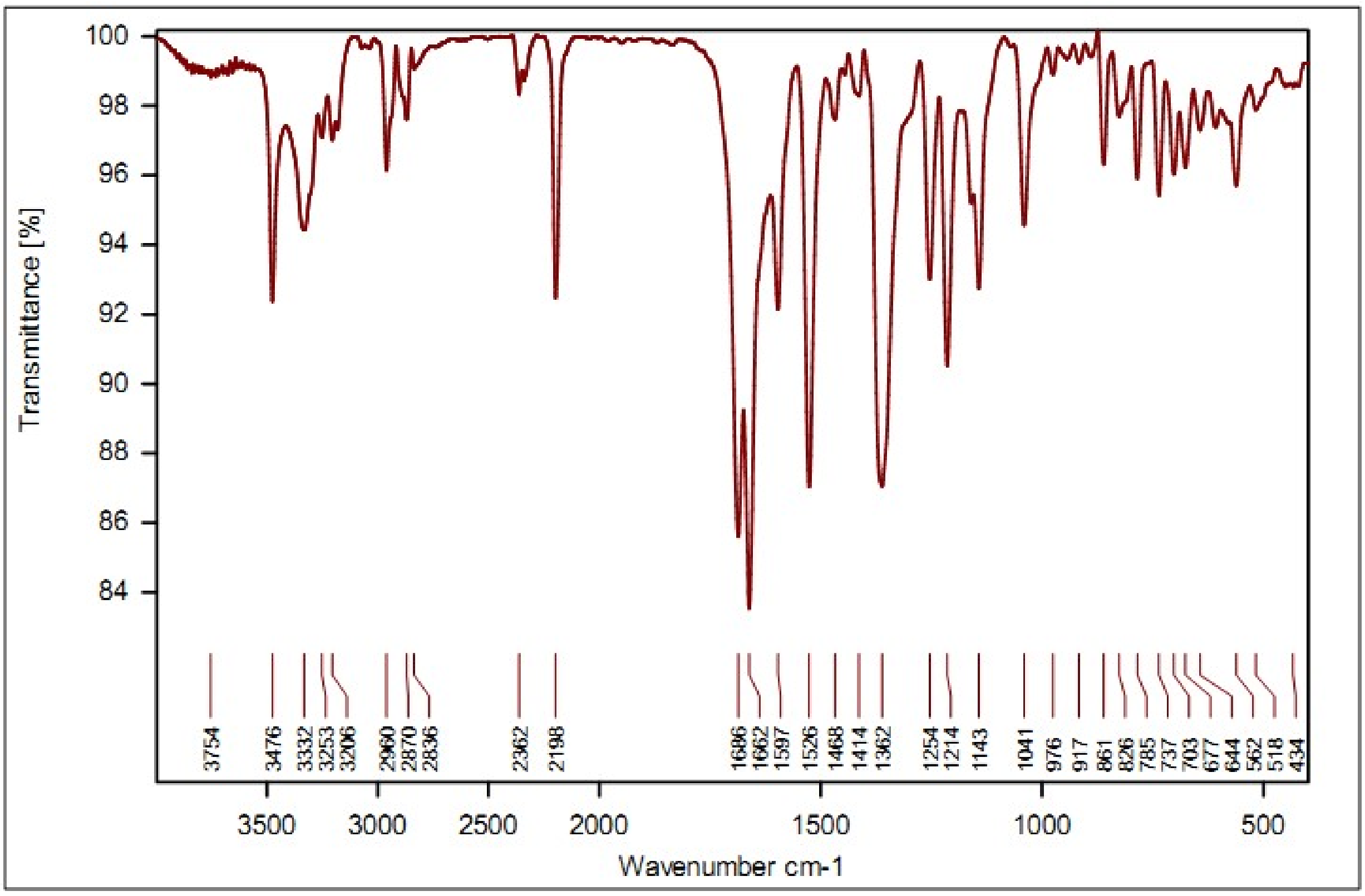

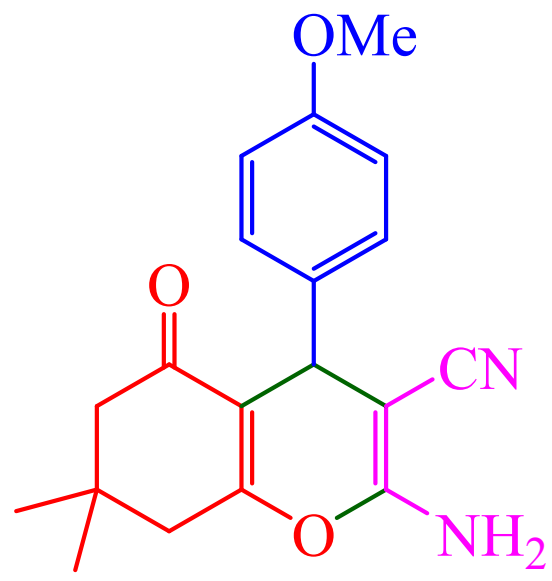

2-amino-4-(4-methoxyphenyl)-7,7-dimethyl-5-oxo-5,6,7,8-tetrahydro-4H-chromene-3-carbonitrile

^{1}H NMR (250 MHz, DMSO$_{d6}$): δ_H= 7.03-6.94 (m, 4H), 6.83-6.79 (m, 2H), 4.09 (s, 1H), 3.68 (s, 3H), 2.48 (s, 2H), 2.26-2.19 (d, *J*= 17.5 Hz, 1H), 2.09-2.03 (d, *J*= 15 Hz, 1H), 1.00 (s, 3H), 0.92 (s, 3H) ppm.

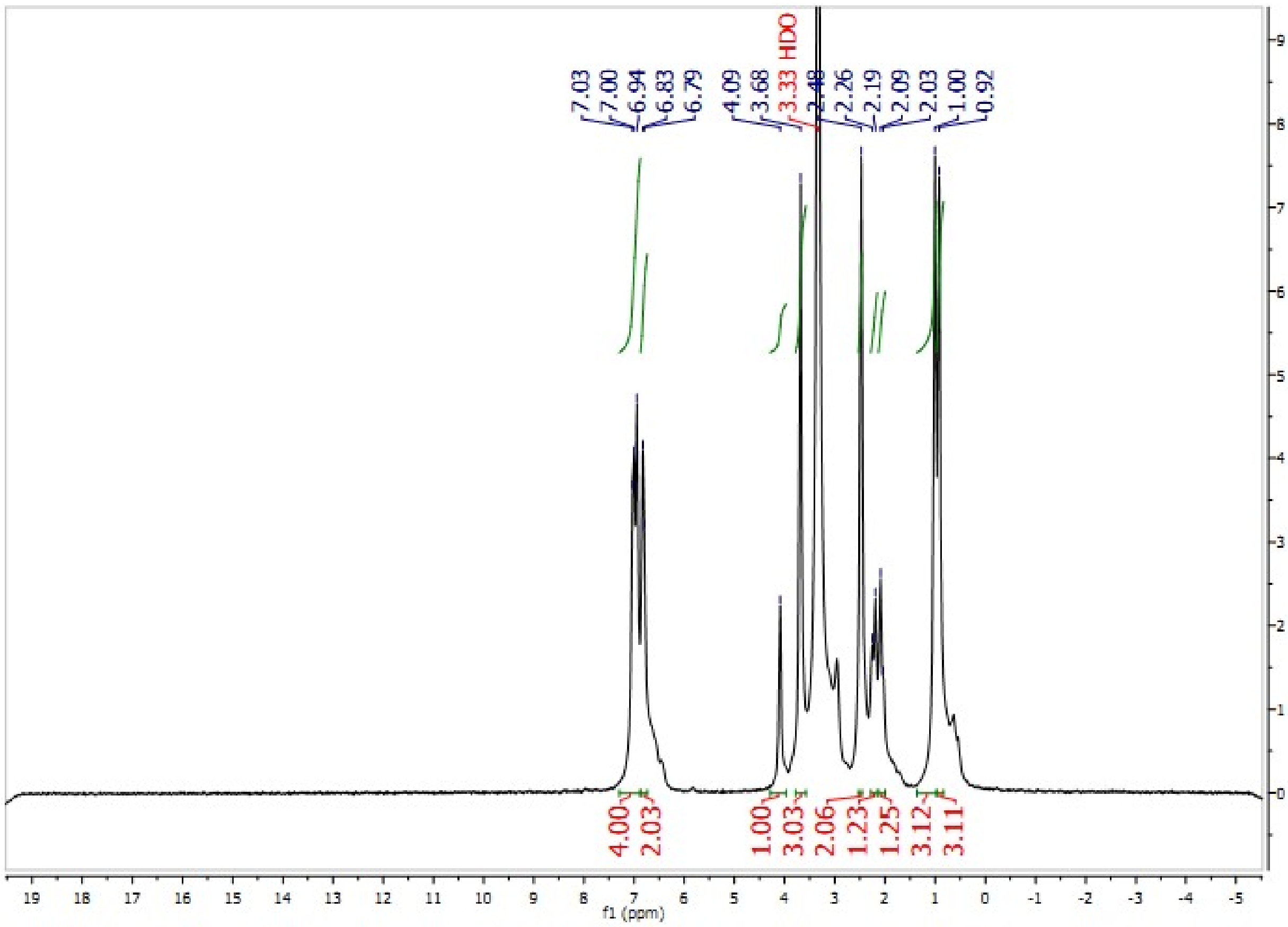

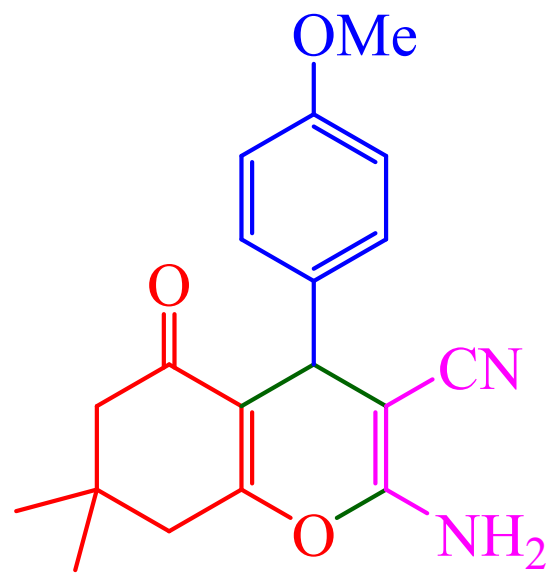

2-amino-4-(4-methoxyphenyl)-7,7-dimethyl-5-oxo-5,6,7,8-tetrahydro-4H-chromene-3-carbonitrile

IR (KBr) cm^{-1}:3374, 3324, 3257, 3186, 3011, 2964, 2895, 2833, 2193, 1685, 1656, 1605, 1509, 1463, 1414, 1369, 1324, 1300, 1252, 1213, 1164, 1139, 1033, 973, 914, 843, 804, 774, 721, 696, 648, 627, 568, 525.

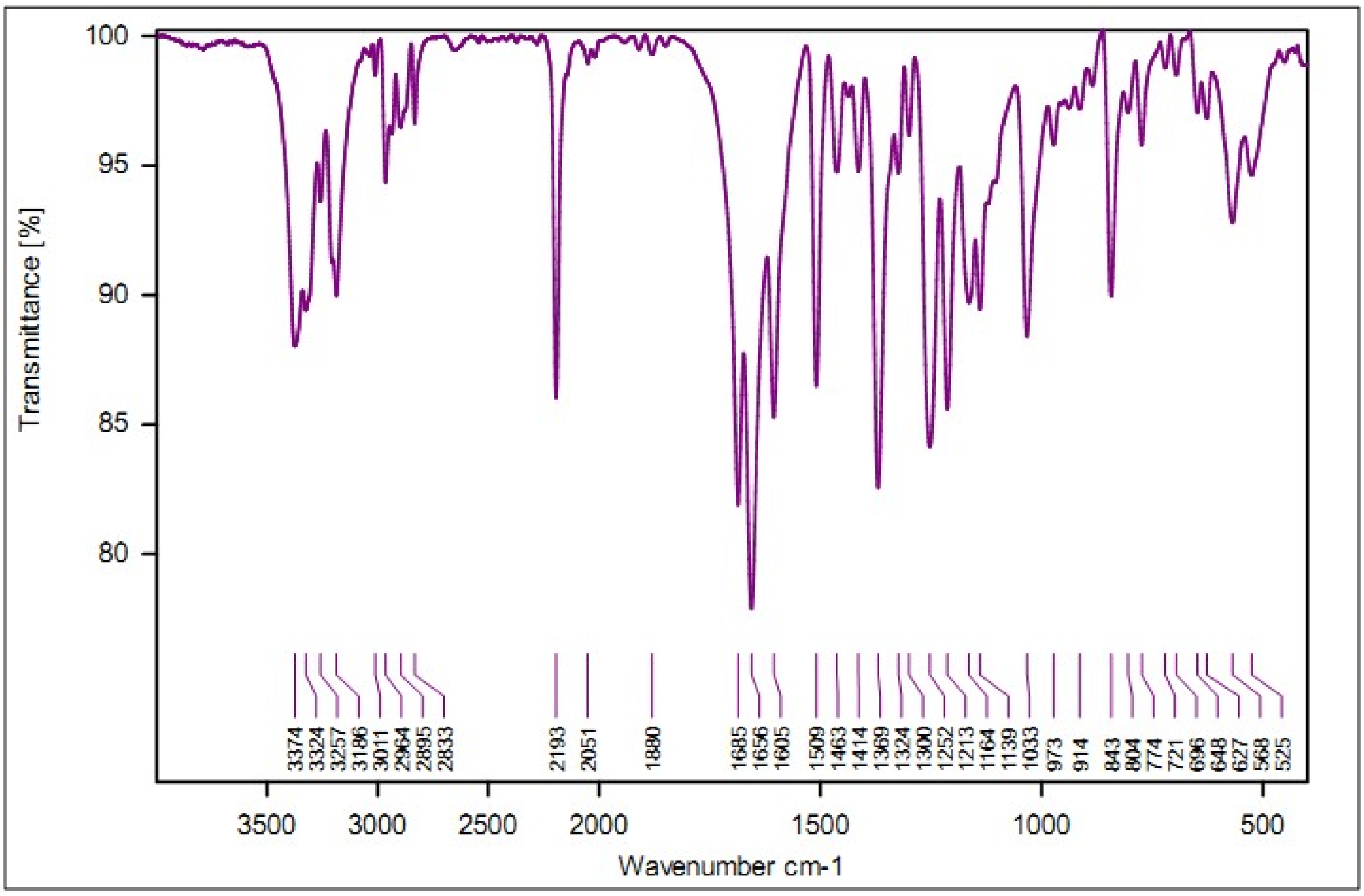

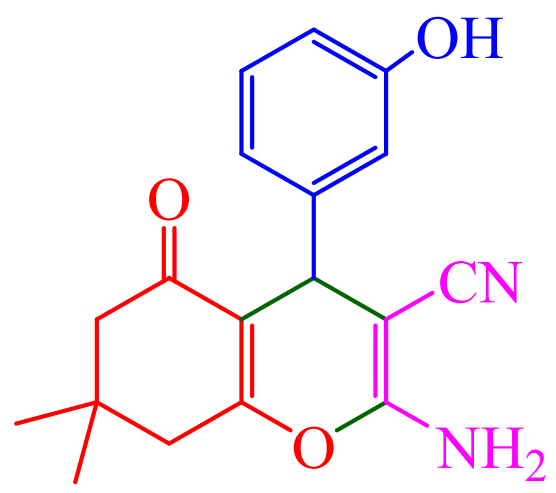

2-amino-4-(3-hydroxyphenyl)-7,7-dimethyl-5-oxo-5,6,7,8-tetrahydro-4H-chromene-3-carbonitrile

^{1}H NMR (250 MHz, DMSO$_{d6}$): δ_H= 9.30 (br, 1H), 7.06-6.96 (m, 3H), 6.54-6.52 (m, 3H), 4.03 (s, 1H), 2.48 (s, 2H), 2.27-2.20 (d, *J*= 17.5 Hz, 1H), 2.11-2.05 (d, *J*= 15 Hz, 1H), 1.01 (s, 3H), 0.94 (s, 3H) ppm.

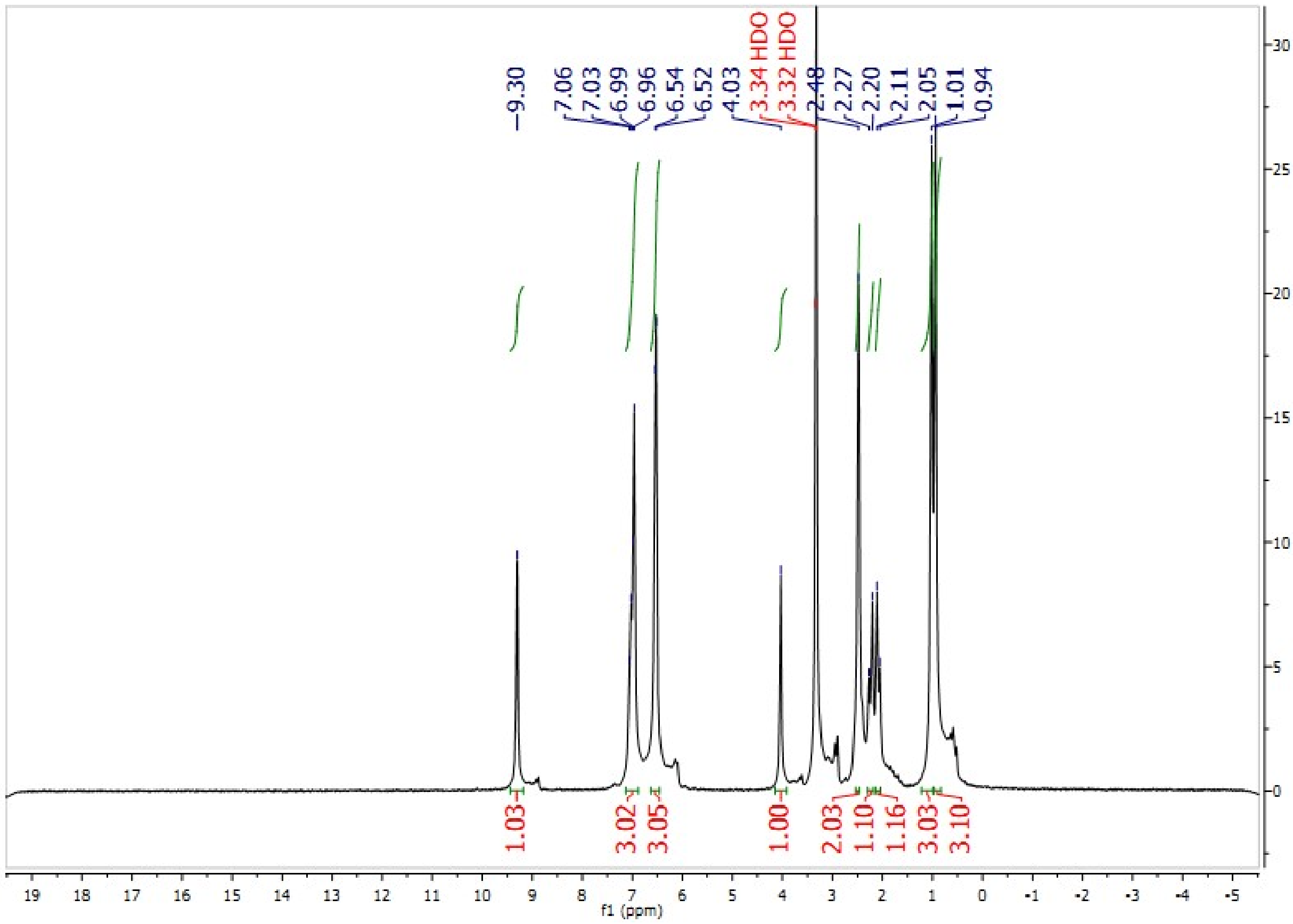

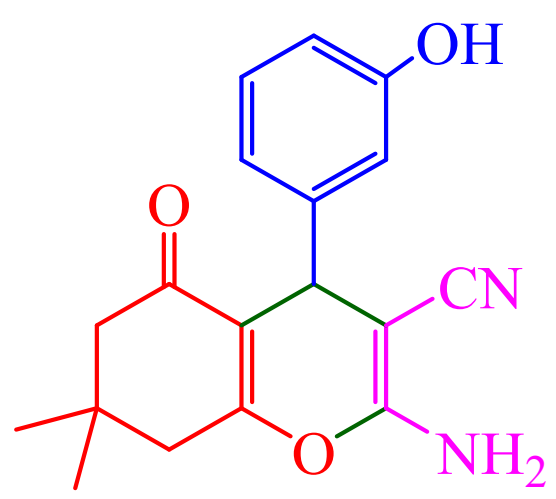

2-amino-4-(3-hydroxyphenyl)-7,7-dimethyl-5-oxo-5,6,7,8-tetrahydro-4H-chromene-3-carbonitrile

^{13}C NMR (100 MHz, $DMSO_{d6}$): δ_C= 195.5, 162.3, 158.5, 157.3, 146.1, 129.1, 119.6, 117.8, 114.1, 113.6, 112.9, 58.6, 50.1, 35.5, 31.7, 28.4, 26.8 ppm.

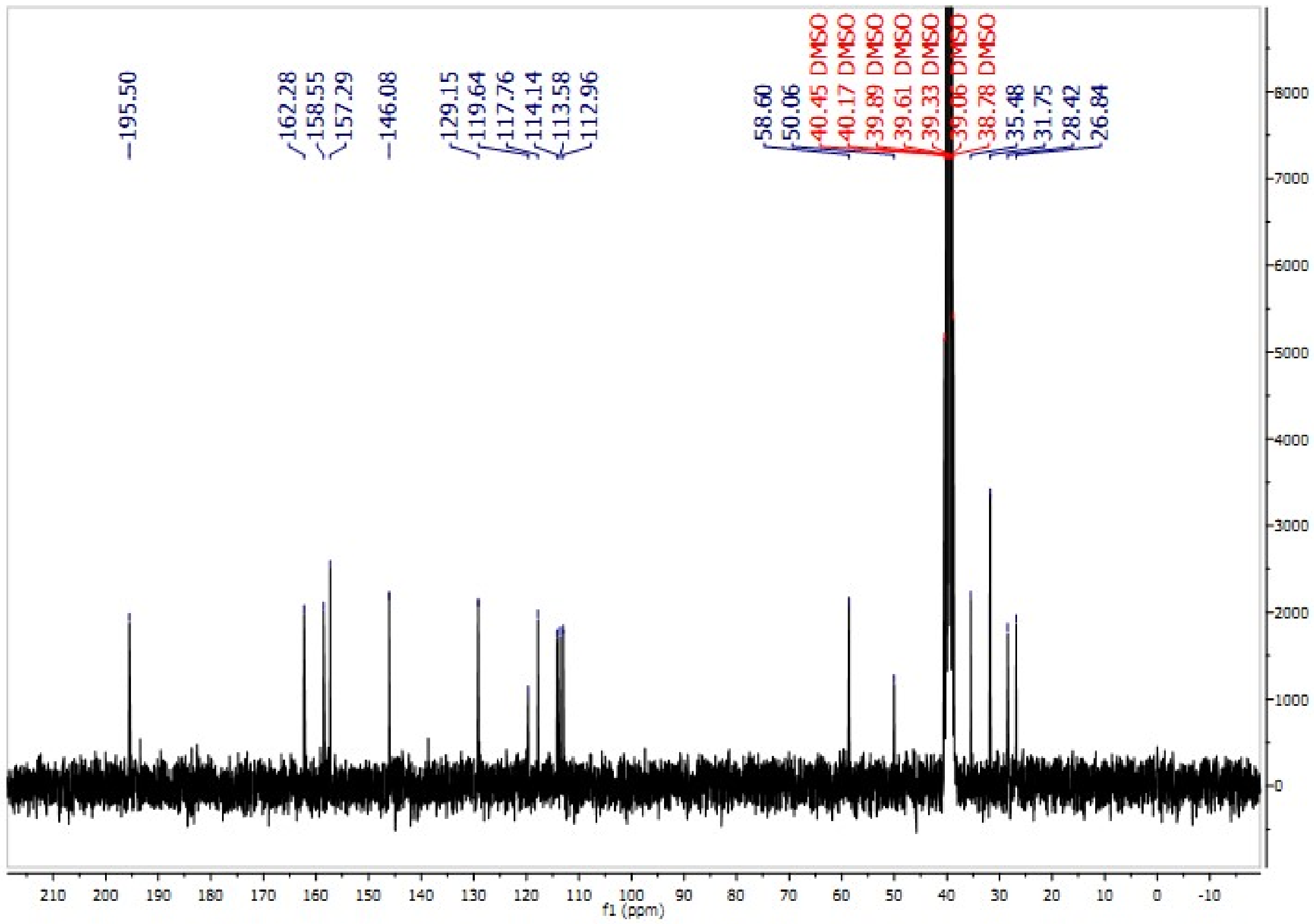

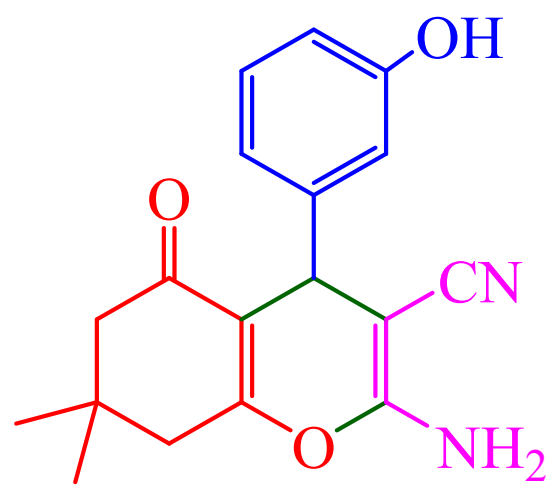

2-amino-4-(3-hydroxyphenyl)-7,7-dimethyl-5-oxo-5,6,7,8-tetrahydro-4H-chromene-3-carbonitrile

IR (KBr) cm^{-1}: 3457, 3312, 3207, 2966, 2879, 2199, 1681, 1643, 1595, 1481, 1375, 1338, 1287, 1257, 1215, 1146, 1079, 1040, 975, 953, 915, 871, 817, 798, 767, 705, 657, 616, 562, 516, 469.

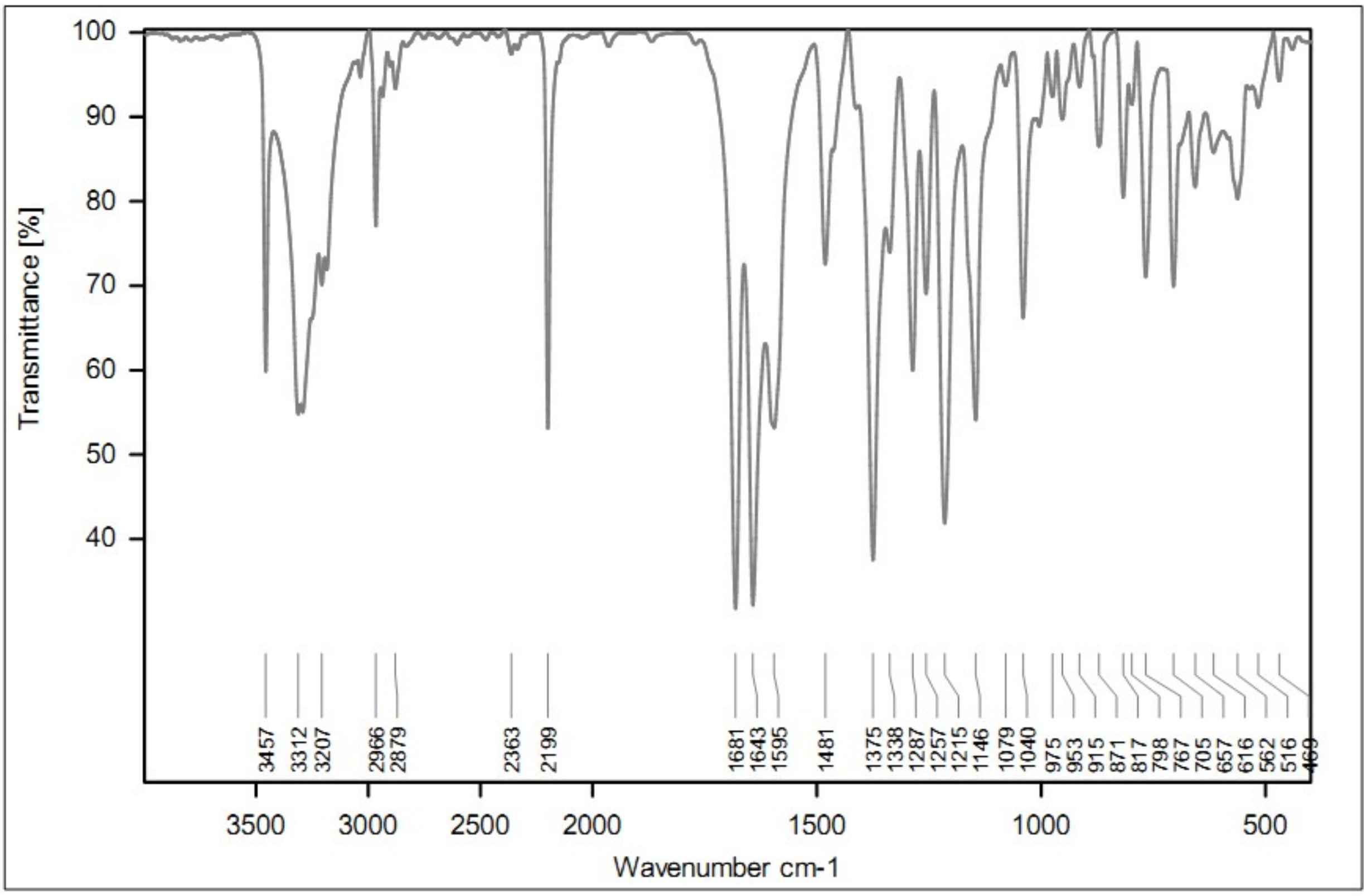

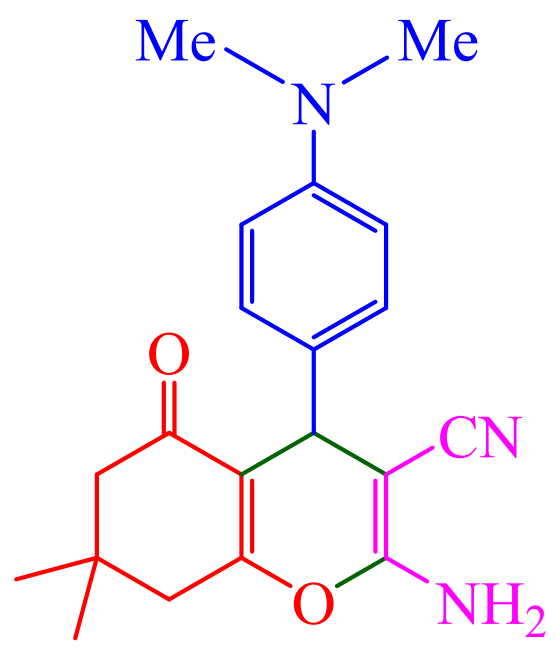

2-amino-4-(4-(dimethylamino)phenyl)-7,7-dimethyl-5-oxo-5,6,7,8-tetrahydro-4H-chromene-3-carbonitrile

^{1}H NMR (250 MHz, DMSO$_{d6}$): δ_H= 6.90-6.89 (m, 4H), 6.61 (br, 2H), 4.01 (s, 1H), 2.47 (s, 2H), 2.25-2.18 (d, *J*= 17.5 Hz, 1H), 2.08-2.01 (d, *J*= 17.5 Hz, 1H), 1.00 (s, 3H), 0.92 (s, 3H) ppm.

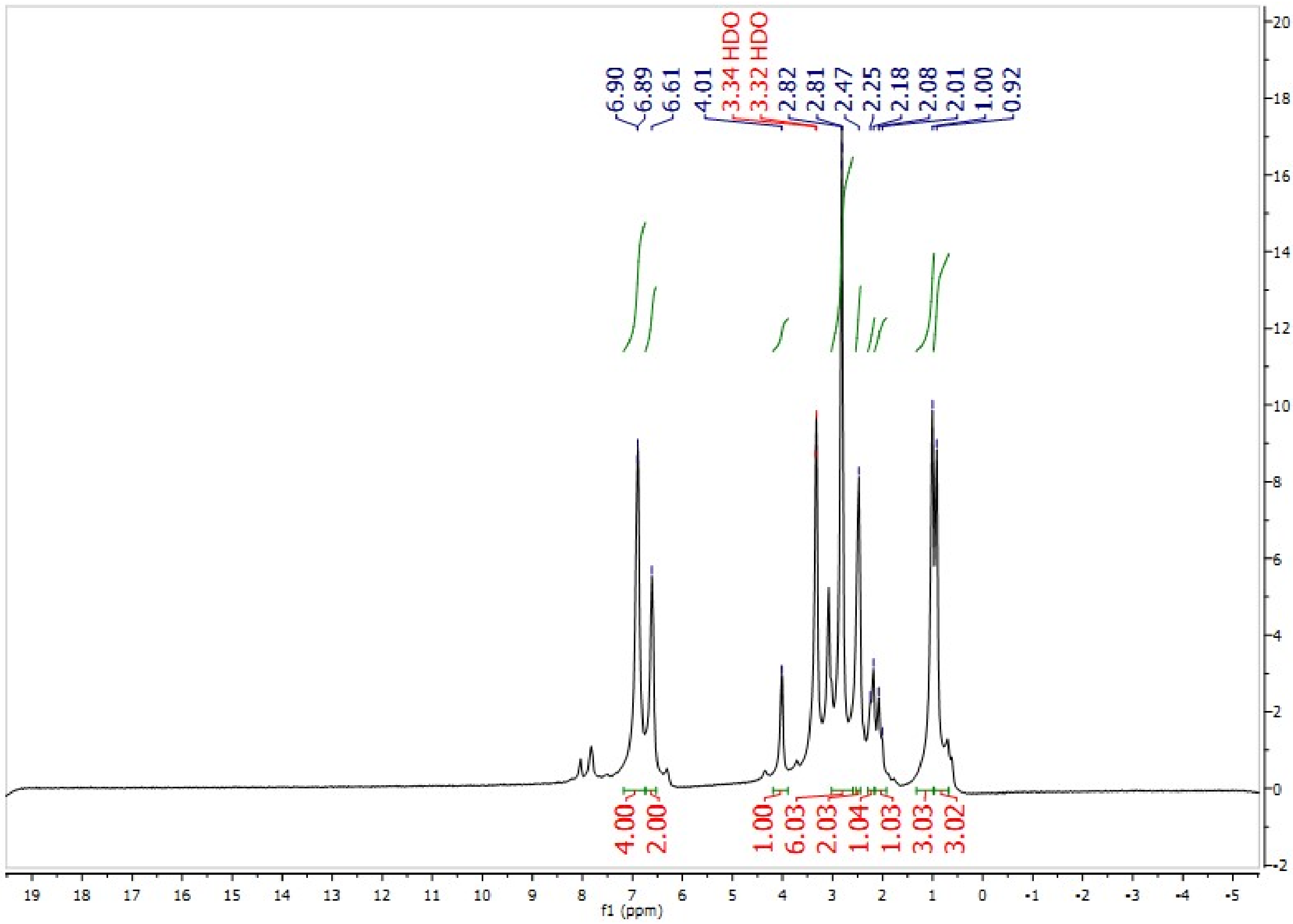

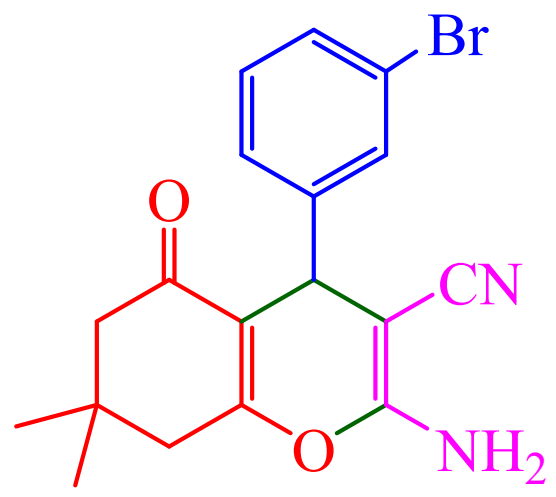

2-amino-4-(3-bromophenyl)-7,7-dimethyl-5-oxo-5,6,7,8-tetrahydro-4H-chromene-3-carbonitrile

^{1}H NMR (250 MHz, DMSO$_{d6}$): δ_H= 7.39-7.22 (m, 3H), 7.15-7.09 (m, 3H), 4.18 (s, 1H), 2.49 (s, 2H), 2.28-2.21 (d, *J*= 17.5 Hz, 1H), 2.13-2.06 (d, *J*= 17.5 Hz, 1H), 1.01 (s, 3H), 0.94 (s, 3H) ppm.

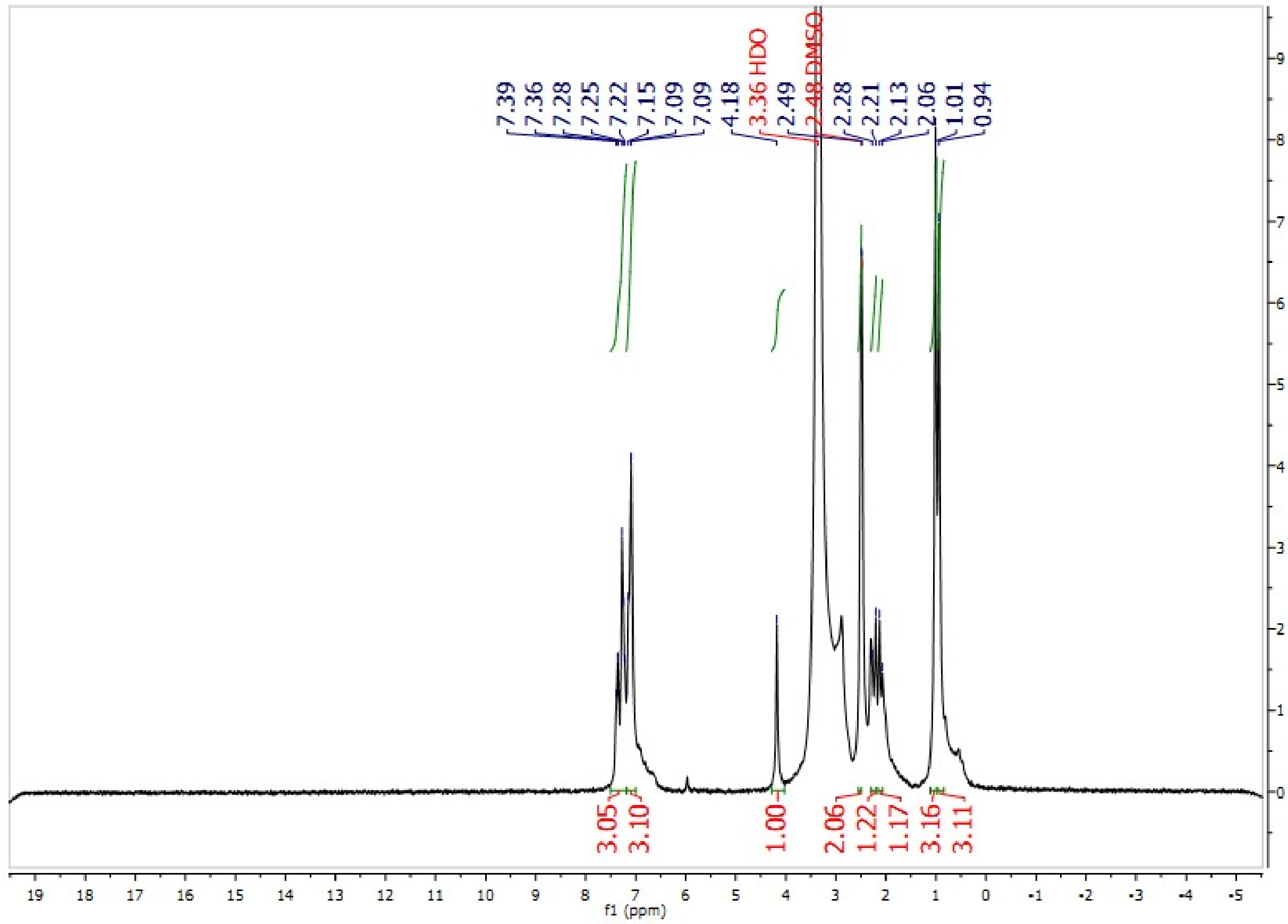

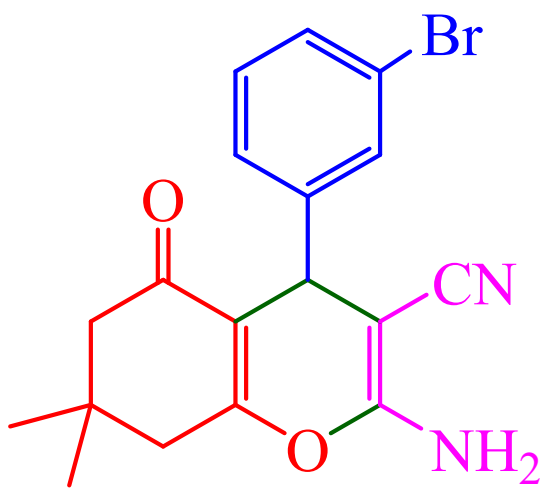

2-amino-4-(3-bromophenyl)-7,7-dimethyl-5-oxo-5,6,7,8-tetrahydro-4H-chromene-3-carbonitrile

IR (KBr) cm^{-1}: 3345, 3259, 3165, 2964, 2192, 1685, 1656, 1604, 1467, 1415, 1371, 1302, 1251, 1215, 1160, 1139, 1068, 1036, 974, 881, 814, 791, 767, 713, 694, 649, 611, 567, 464, 435.

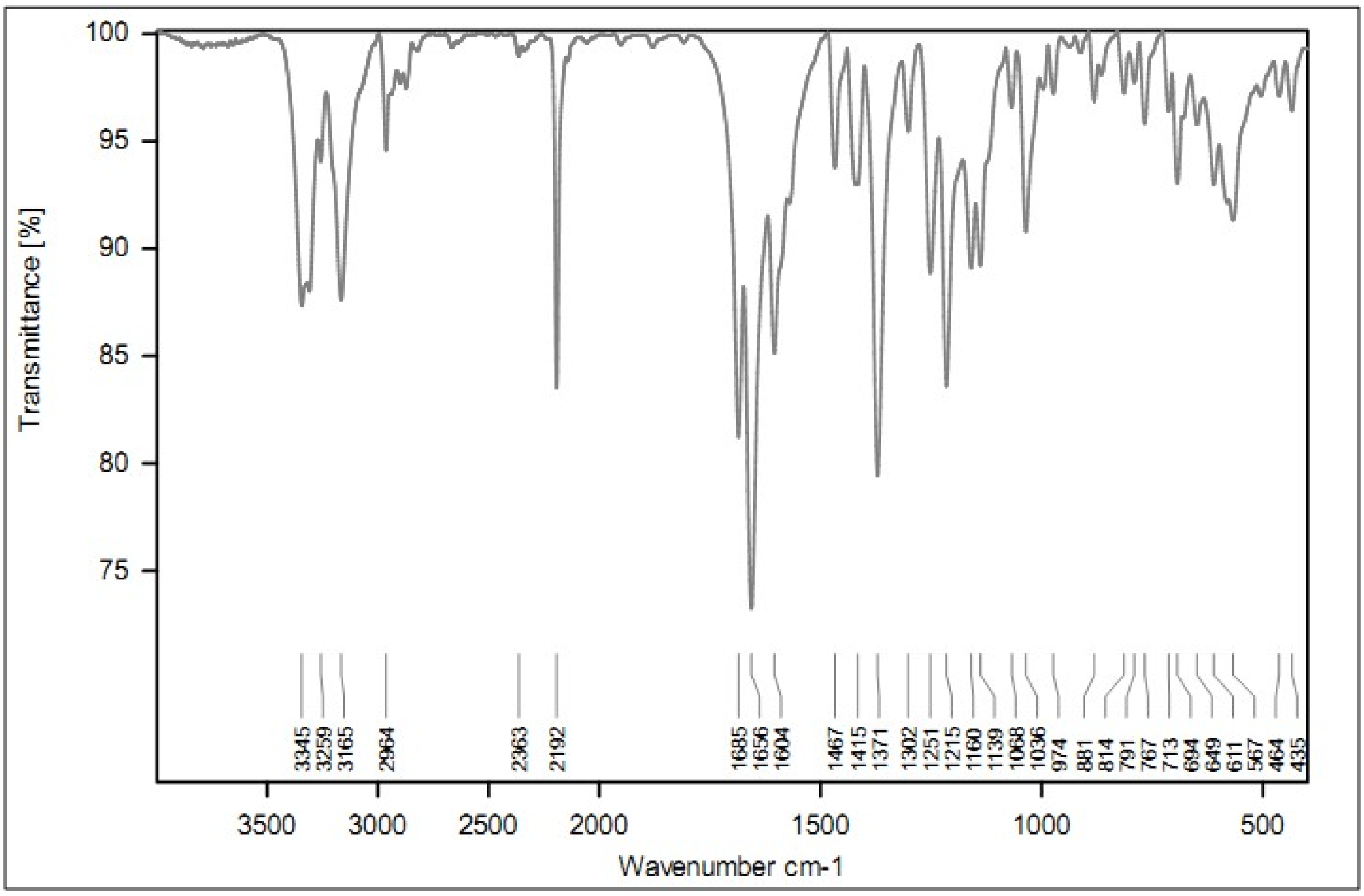

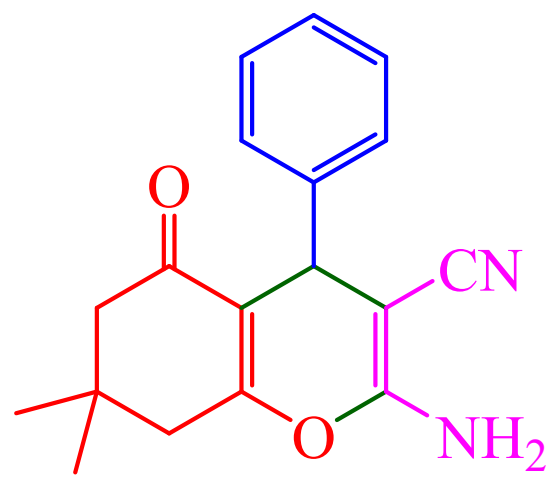

2-amino-7,7-dimethyl-5-oxo-4-phenyl-5,6,7,8-tetrahydro-4H-chromene-3-carbonitrile

^{1}H NMR (250 MHz, DMSO$_{d6}$): δ_H= 7.27-7.24 (d, *J*= 7.5 Hz, 2H), 7.19-7.10 (m, 3H), 7.00 (br, 2H), 4.15 (s, 1H), 2.50 (s, 2H), 2.27-2.21 (d, *J*= 15 Hz, 1H), 2.11-2.05 (d, *J*= 15 Hz, 1H), 1.02 (s, 3H), 0.94 (s, 3H) ppm.

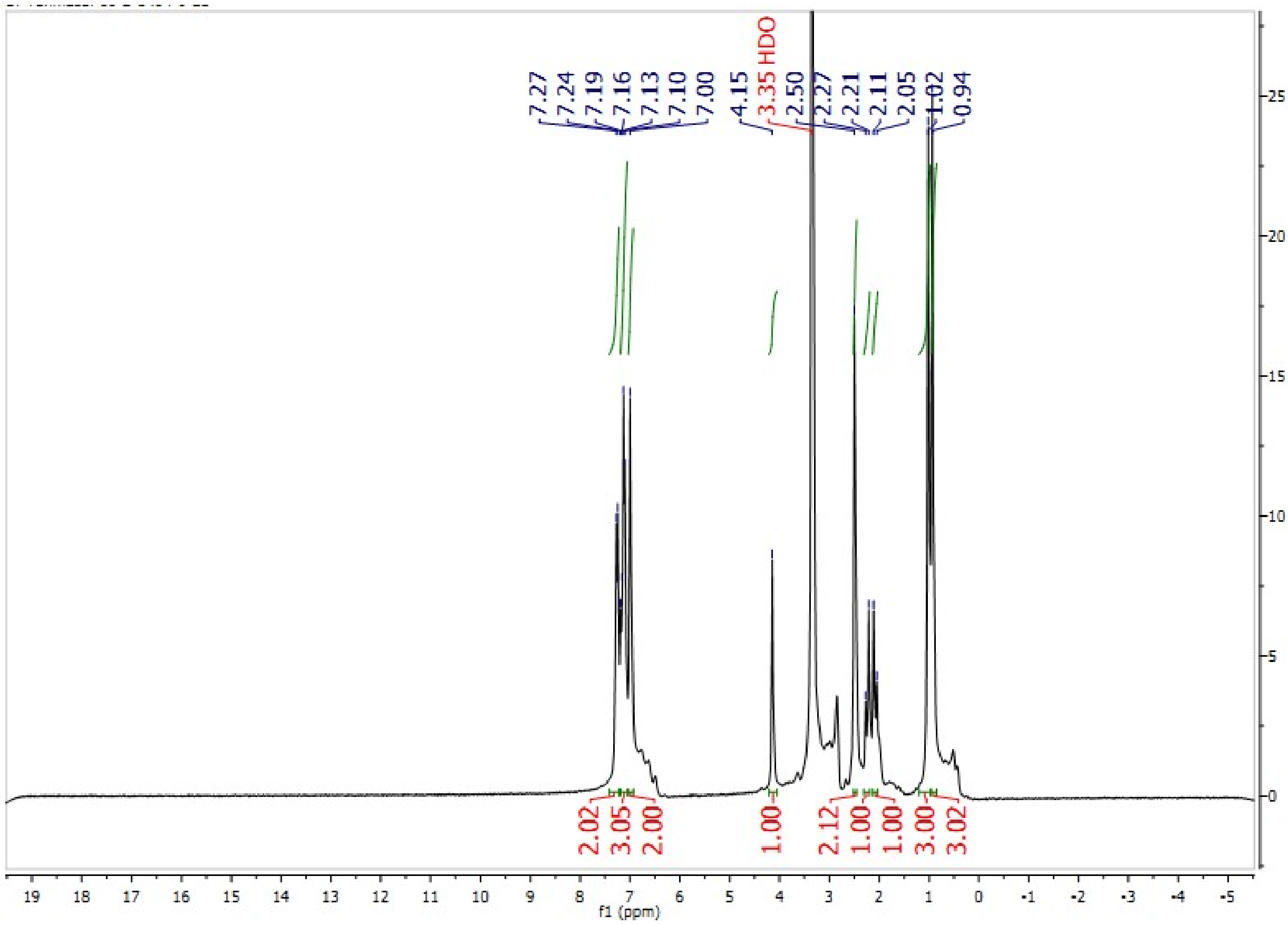

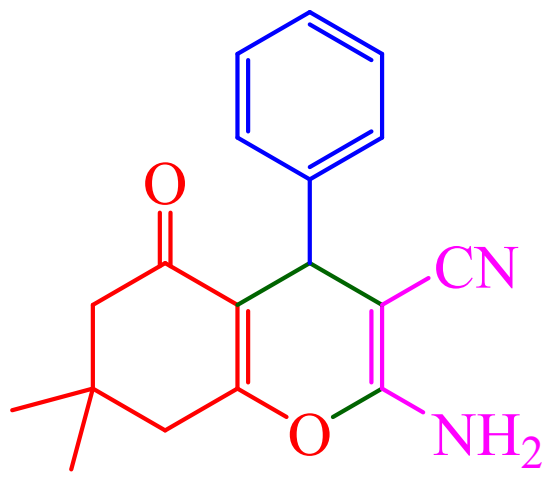

2-amino-7,7-dimethyl-5-oxo-4-phenyl-5,6,7,8-tetrahydro-4H-chromene-3-carbonitrile

IR (KBr) cm^{-1}: 3396, 3325, 3252, 3212, 3028, 2964, 2883, 2825, 2199, 1682, 1660, 1603, 1492, 1452, 1413, 1370, 1249, 1214, 1159, 1138, 1035, 972, 887, 838, 815, 786, 737, 696, 652, 580, 560, 530, 495, 422.

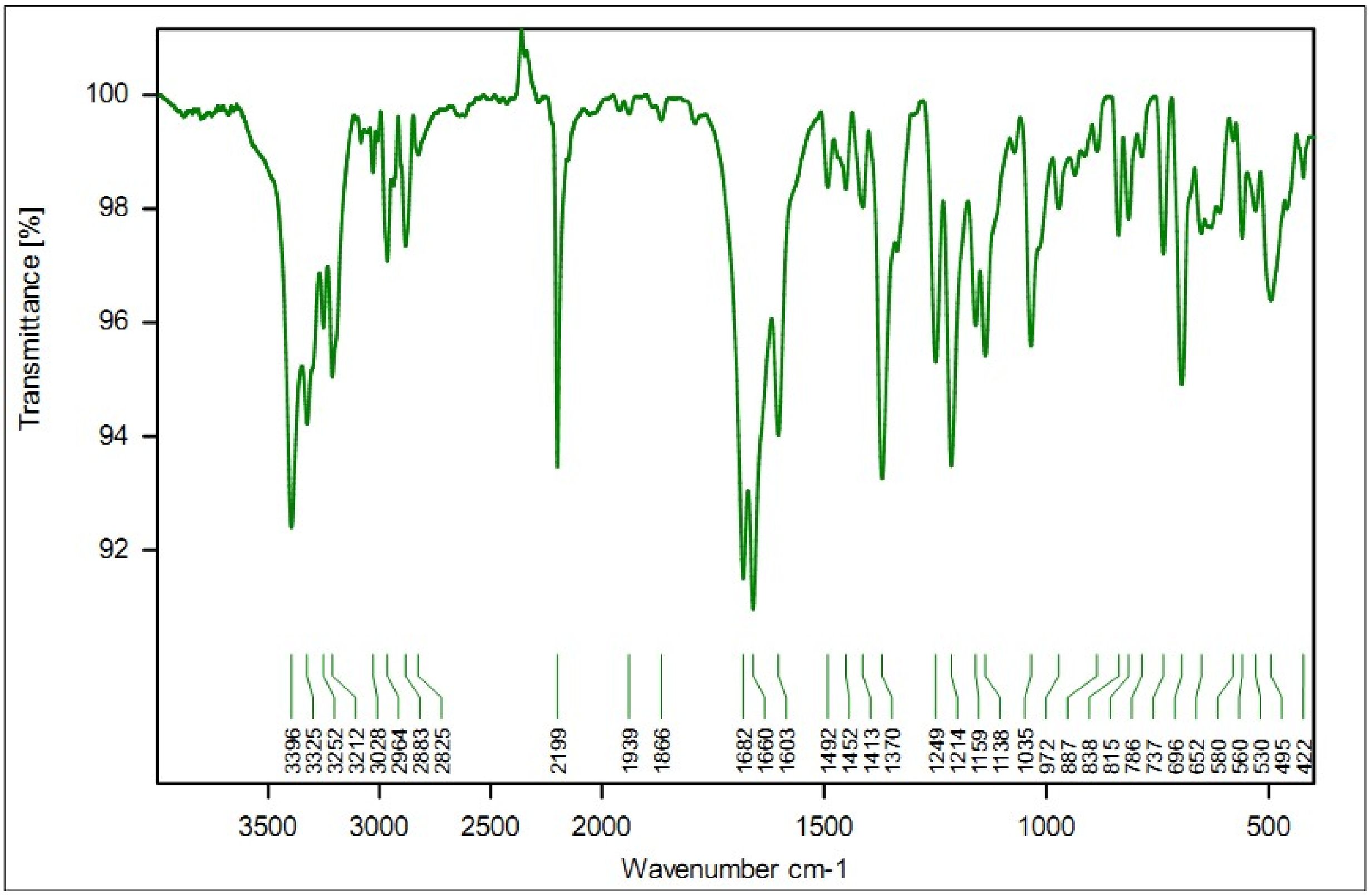

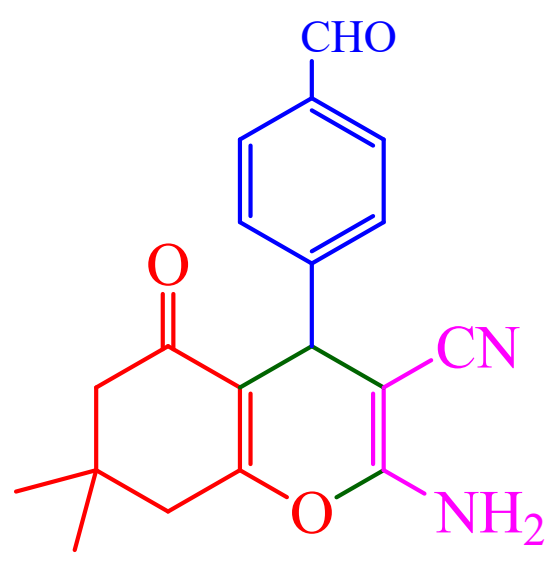

2-amino-4-(4-formylphenyl)-7,7-dimethyl-5-oxo-5,6,7,8-tetrahydro-4H-chromene-3-carbonitrile

^{1}H NMR (300 MHz, $DMSO_{d6}$): δ_H= 9.93 (s, 1H), 7.84-7.81 (d, *J*= 9 Hz, 2H), 7.37-7.35 (d, *J*= 6 Hz, 2H), 7.14 (br, 2H), 4.27 (s, 1H), 2.52 (s, 2H), 2.27-2.22 (d, *J*= 15 Hz, 1H), 2.11-2.06 (d, *J*= 15 Hz, 1H), 1.02 (s, 3H), 0.94 (s, 3H) ppm.

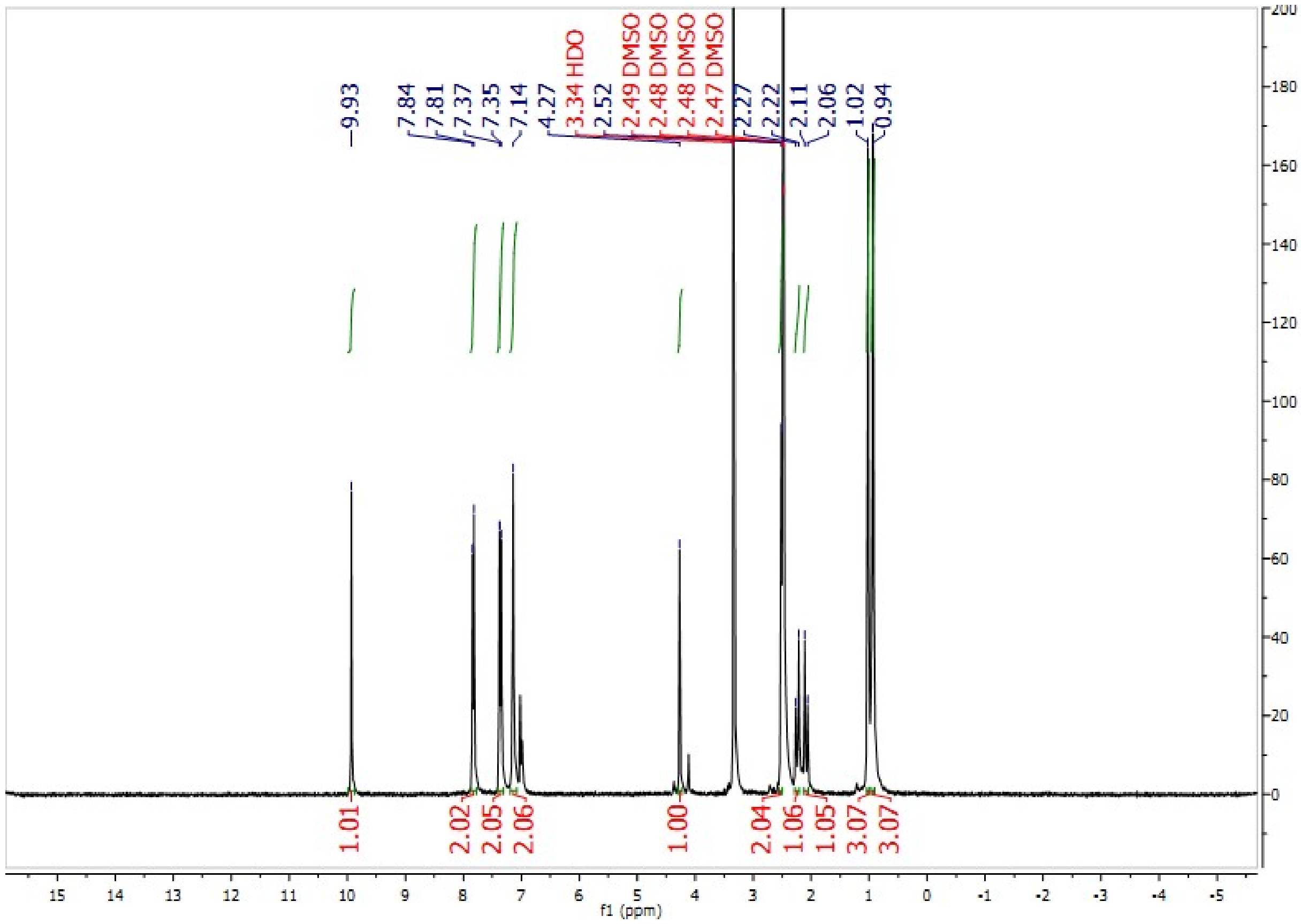

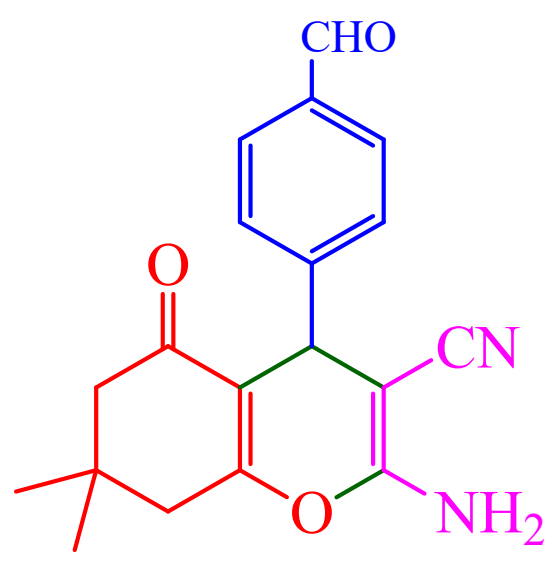

2-amino-4-(4-formylphenyl)-7,7-dimethyl-5-oxo-5,6,7,8-tetrahydro-4H-chromene-3-carbonitrile

IR (KBr) cm^{-1}: 3408, 3332, 3257, 3212, 2962, 2934, 2872, 2193, 1686, 1602, 1575, 1465, 1414, 1396, 1367, 1321, 1250, 1213, 1144, 1040, 973, 916, 851, 819, 796, 773, 694, 623, 562, 507.

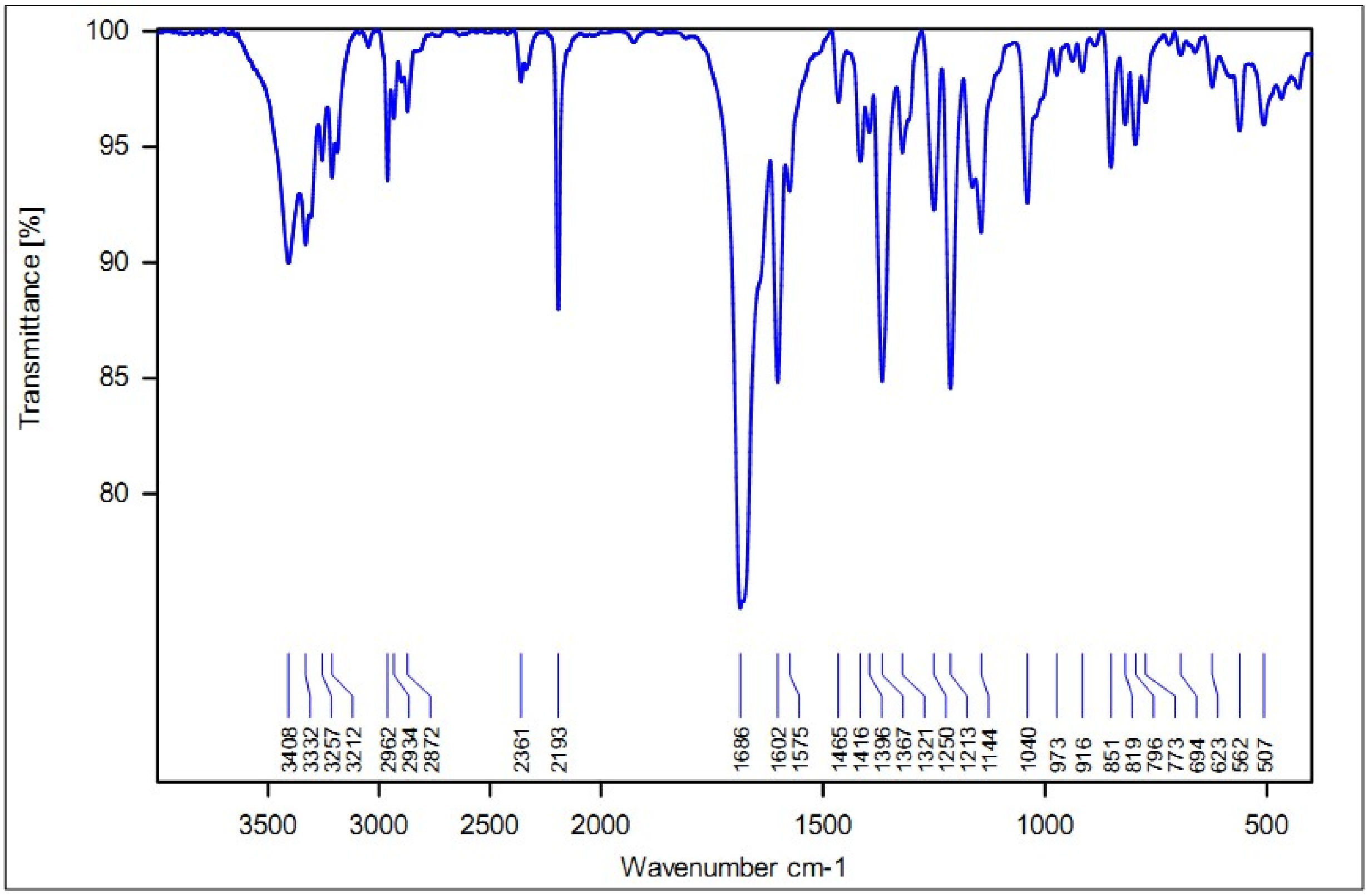

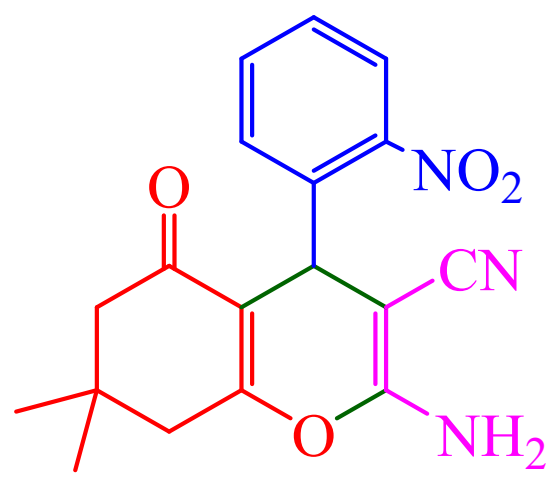

2-amino-7,7-dimethyl-4-(2-nitrophenyl)-5-oxo-5,6,7,8-tetrahydro-4H-chromene-3-carbonitrile

^{1}H NMR (300 MHz, $DMSO_{d6}$): δ_H= 8.08-8.05 (d, *J*= 9 Hz, 1H), 7.95 (s, 1H), 7.66-7.57 (m, 2H), 7.20 (br, 2H), 4.40 (s, 1H), 2.53 (s, 2H), 2.28-2.23 (d, *J*= 15 Hz, 1H), 2.12-2.07 (d, *J*= 15 Hz, 1H), 1.03 (s, 3H), 0.94 (s, 3H) ppm.

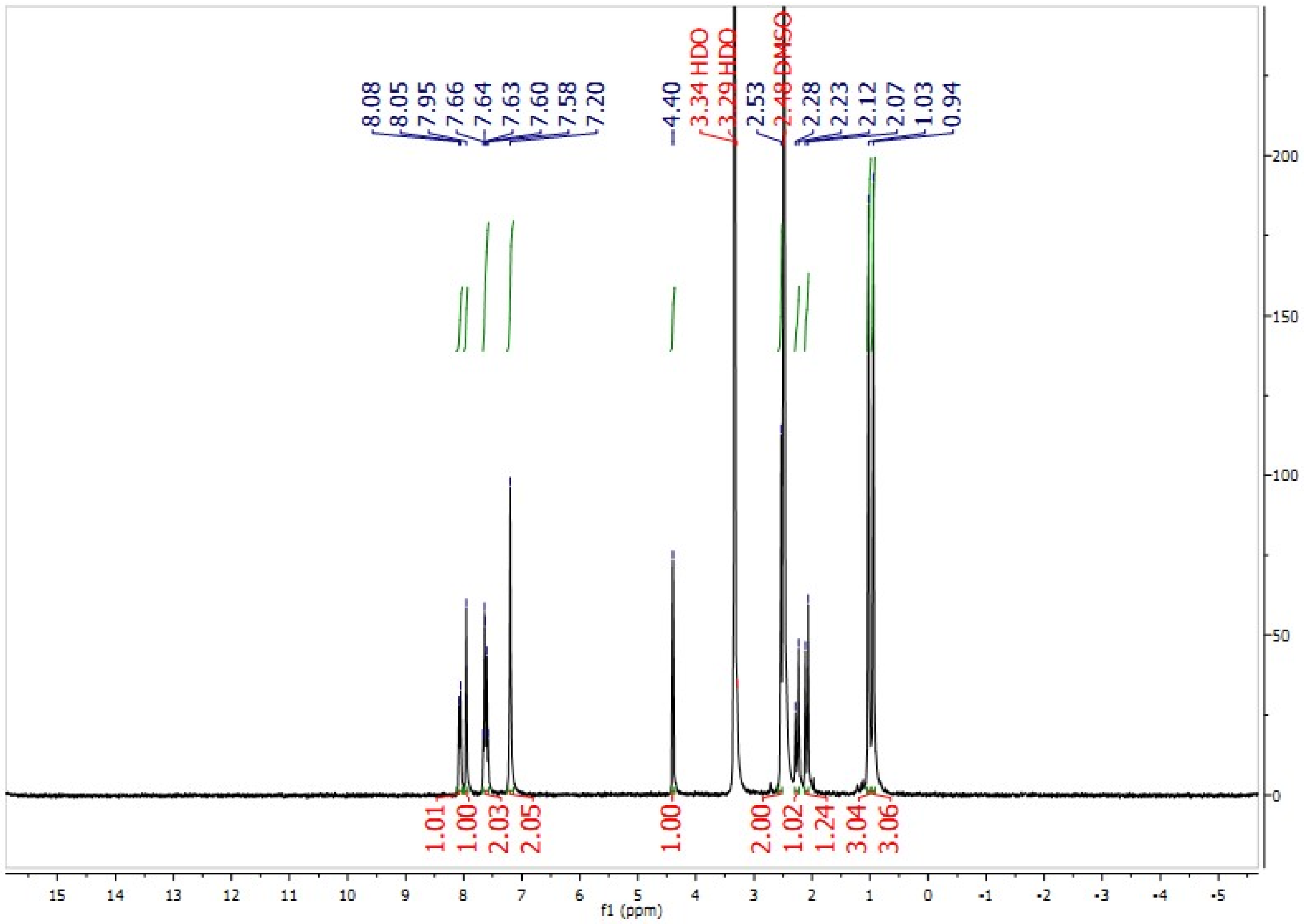

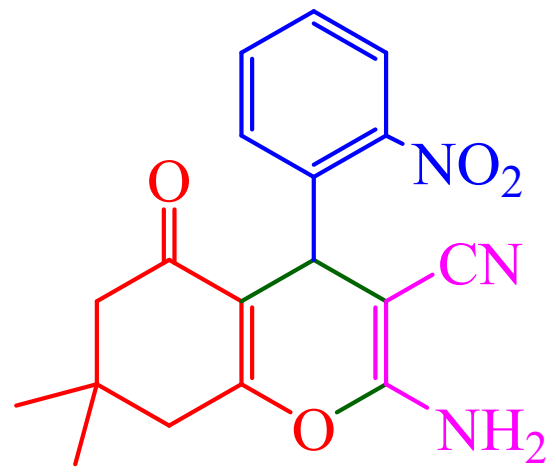

2-amino-7,7-dimethyl-4-(2-nitrophenyl)-5-oxo-5,6,7,8-tetrahydro-4H-chromene-3-carbonitrile

^{13}C NMR (100 MHz, $DMSO_{d6}$): δ_C= 195.6, 163.1, 158.7, 147.8, 146.9, 134.1, 129.9, 121.7, 121.6, 119.2, 111.9, 57.4, 49.9, 35.5, 31.8, 28.3, 26.8 ppm.

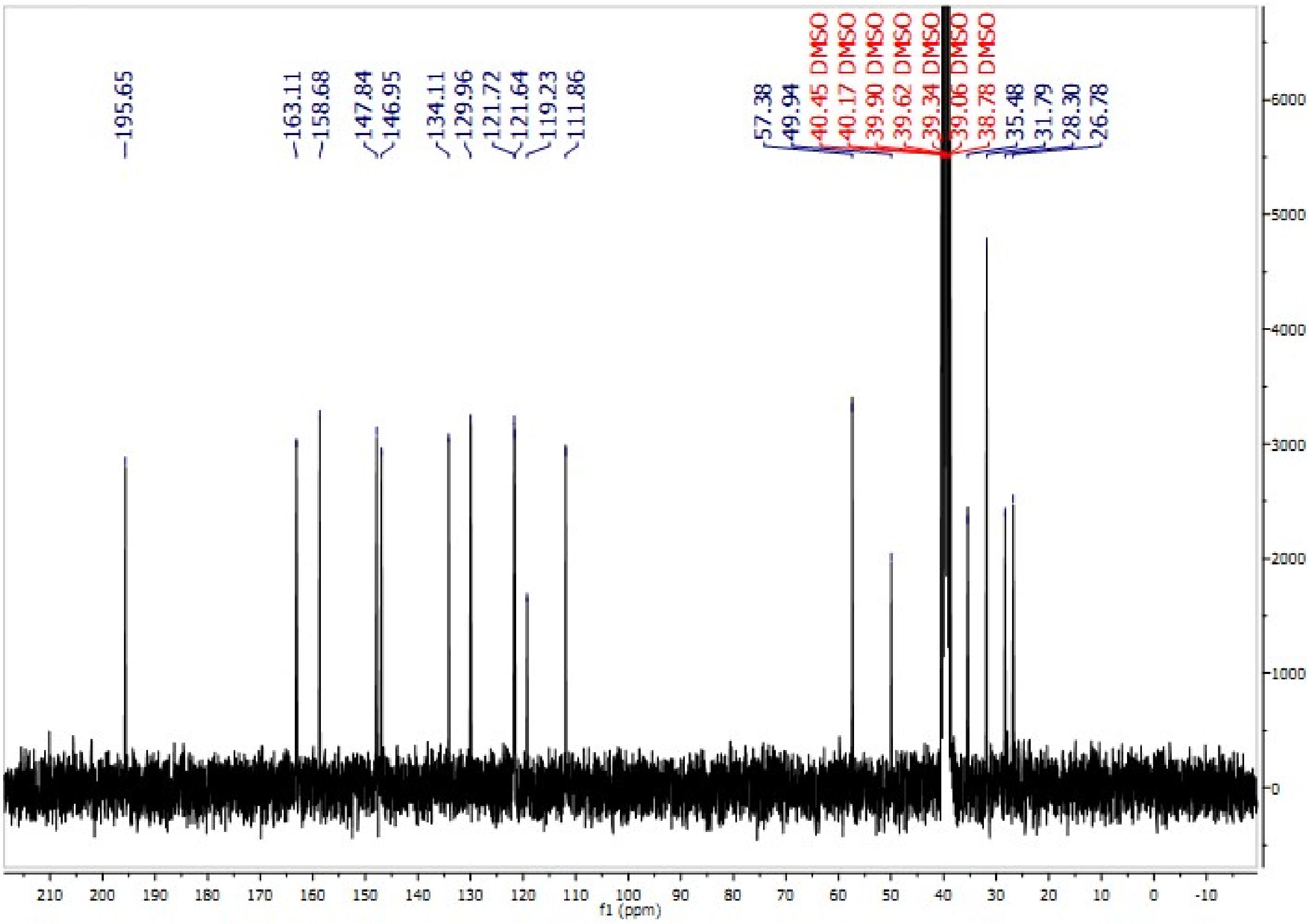

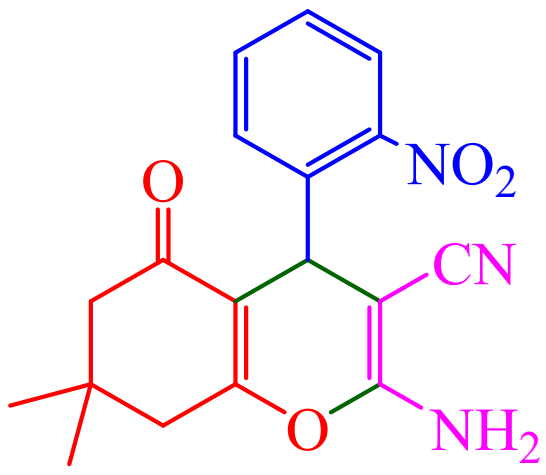

2-amino-7,7-dimethyl-4-(2-nitrophenyl)-5-oxo-5,6,7,8-tetrahydro-4H-chromene-3-carbonitrile

IR (KBr) cm^{-1}: 3471, 3334, 3256, 3211, 3077, 2961, 2870, 2194, 1689, 1663, 1598, 1525, 1468, 1447, 1412, 1360, 1254, 1212, 1162, 1143, 1042, 977, 946, 917, 861, 827, 784, 735, 699, 675, 644, 610, 561, 514, 455.

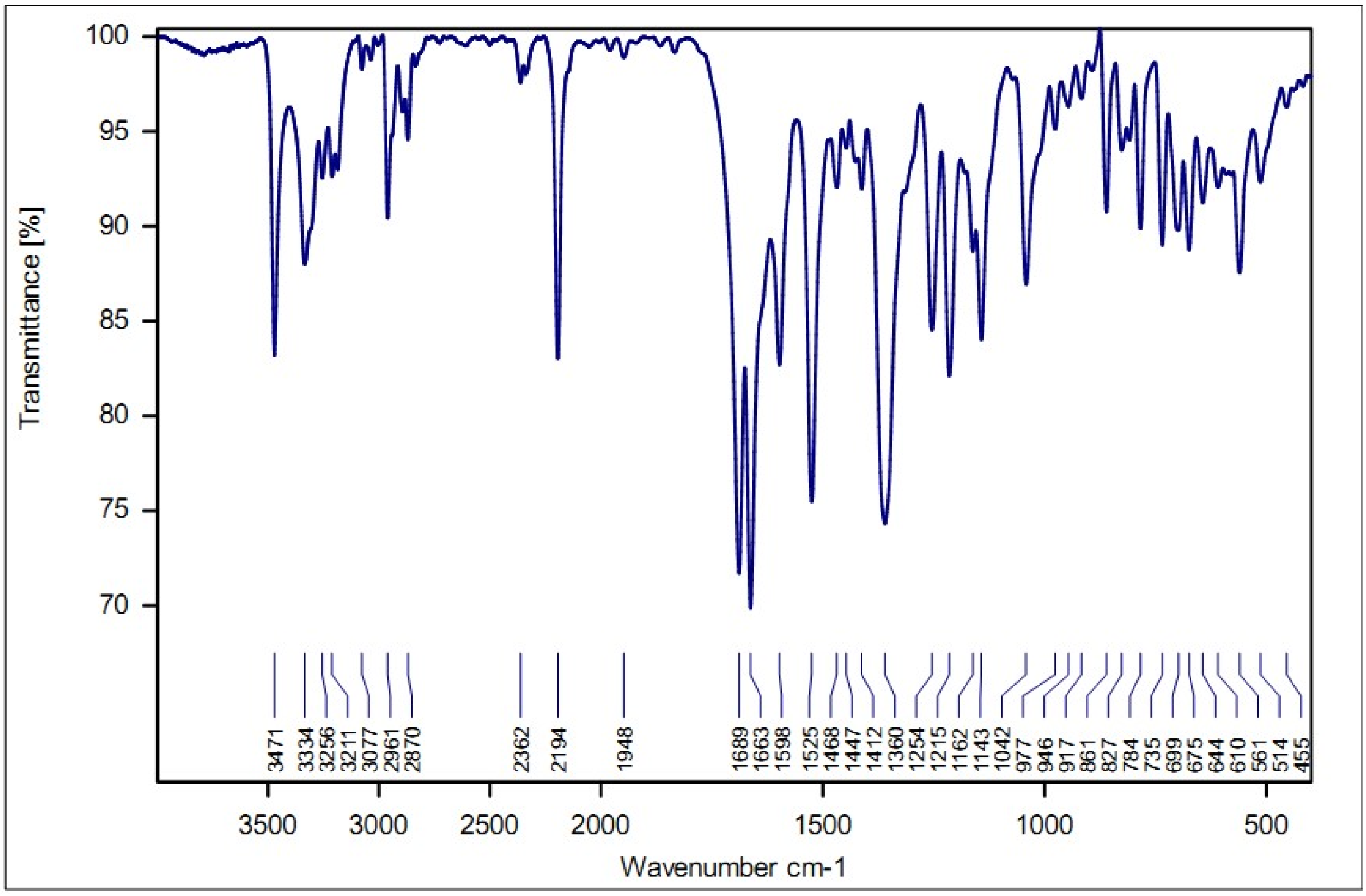

Supplement: Supplementary file 1 [file RA-016-D6RA02796B-s001.pdf]
